# Regional paleoclimates and local consequences: Integrating GIS analysis of diachronic settlement patterns and process-based agroecosystem modeling of potential agricultural productivity in Provence (France)

**DOI:** 10.1371/journal.pone.0207622

**Published:** 2018-12-12

**Authors:** Daniel A. Contreras, Eneko Hiriart, Alberte Bondeau, Alan Kirman, Joël Guiot, Loup Bernard, Romain Suarez, Sander Van Der Leeuw

**Affiliations:** 1 Institut Méditerranéen de Biodiversité et d’Ecologie marine et continentale (IMBE), Aix-Marseille Université, CNRS, IRD, Avignon Université, Aix-en-Provence, France; 2 Groupement de recherche en économie quantitative d’Aix-Marseille (GREQAM), Aix-Marseille Université, Aix-en-Provence, France; 3 Department of Anthropology, University of Maryland, College Park, Maryland, United States of America; 4 Aix-Marseille University, CNRS, Ministère de la Culture, MMSH, Centre Camille Julian, Maison Méditerranéenne des Sciences de l’Homme, Aix-en-Provence, France; 5 CRP2A-IRAMAT (UMR 5060), Université Bordeaux Montaigne, Bordeaux, France; 6 CAMS-EHESS, Ecole des Hautes Etudes en Sciences Sociales and Aix-Marseille Université, Aix-en-Provence, France; 7 Aix-Marseille Université, CNRS, IRD, INRA, Coll France, CEREGE, Aix-en-Provence, France; 8 Université de Strasbourg, Université de Haute-Alsace, CNRS, Archimède UMR, Strasbourg, France; 9 LabEx OT-Med, Aix-Marseille Université, Aix-en-Provence, France; 10 Schools of Sustainability and Human Evolution and Social Change, Arizona State University, Tempe, Arizona, United States of America; New York State Museum, UNITED STATES

## Abstract

Holocene climate variability in the Mediterranean Basin is often cited as a potential driver of societal change, but the mechanisms of this putative influence are generally little explored. In this paper we integrate two tools–agro-ecosystem modeling of potential agricultural yields and spatial analysis of archaeological settlement pattern data–in order to examine the human consequences of past climatic changes. Focusing on a case study in Provence (France), we adapt an agro-ecosystem model to the modeling of potential agricultural productivity during the Holocene. Calibrating this model for past crops and agricultural practices and using a downscaling approach to produce high spatiotemporal resolution paleoclimate data from a Mediterranean Holocene climate reconstruction, we estimate realistic potential agricultural yields under past climatic conditions. These serve as the basis for spatial analysis of archaeological settlement patterns, in which we examine the changing relationship over time between agricultural productivity and settlement location. Using potential agricultural productivity (PAgP) as a measure of the human consequences of climate changes, we focus on the relative magnitudes of 1) climate-driven shifts in PAgP and 2) the potential increases in productivity realizable through agricultural intensification. Together these offer a means of assessing the scale and mechanisms of the vulnerability and resilience of Holocene inhabitants of Provence to climate change. Our results suggest that settlement patterns were closely tied to PAgP throughout most of the Holocene, with the notable exception of the period from the Middle Bronze Age through the Early Iron Age. This pattern does not appear to be linked to any climatically-driven changes in PAgP, and conversely the most salient changes in PAgP during the Holocene cannot be clearly linked to any changes in settlement pattern. We argue that this constitutes evidence that vulnerability and resilience to climate change are strongly dependent on societal variables.

## 1. Introduction

Past climatic changes are often cited as drivers of societal change in the Mediterranean Basin (e.g., [1–7]). While Holocene climate variability in the region is certainly sufficient to raise questions about if and how inhabitants responded, the mechanisms of this putative influence are generally little explored, reflecting the general interdisciplinary challenge of integrating paleoclimatic and archaeological data and models that are of varying and often incommensurate scales and resolutions (cf. [8]). In this paper we integrate two tools–agro-ecosystem modeling of potential yields and spatial analysis of archaeological settlement pattern data–in order to examine the human consequences of past climatic changes. Focusing on a case study in Provence (France), we adapt the agro-ecosystem model LPJmL (the Lund-Potsdam-Jena-managed-land model, [9]) to model potential agricultural productivity during the Holocene. Calibrating this model for past crops and agricultural practices (see [10]) and using a downscaling approach to produce high spatiotemporal resolution paleoclimate data (see [11]), we estimate reasonable potential agricultural yields under past climatic conditions. These serve as the basis for spatial analysis of archaeological settlement pattern data derived from *Patriarche* (the French national archaeological atlas, a continuously updated database that integrates excavation and survey data from diverse sources: http://www.culturecommunication.gouv.fr/Politiques-ministerielles/Archeologie/Etude-recherche/Carte-archeologique-nationale),which we use to examine the changing relationship over time between potential agricultural productivity and settlement location.

Potential agricultural productivity (PAgP) is strongly related to climatic factors (particularly, in a Mediterranean climate, precipitation) while also more directly relevant to human activity than raw climatic variables. Although PAgP is largely a function of these climatic variables, using a derived variable allows moving beyond simplistic threshold approaches to more realistic assessments of the spatiotemporally variable and not necessarily linear consequences of climate change. By exploring if and how settlement location was related to PAgP in different periods of the Holocene, we approach the problem of relating diachronic cultural patterns to climatic changes empirically. Using a site location analysis approach in which we statistically summarize geographic variables and PAgP within buffers around archaeologically-documented settlements, we explore a) how settlement locations relate to spatially- and temporally-variable PAgP, b) whether changes over time in PAgP had any apparent effect on settlement locations, and c) the implications of the reconstructed variability in PAgP and past agricultural practices for understanding the vulnerability and resilience of Holocene Mediterranean populations to climate changes.

Site location analysis and related approaches have a long history in archaeology (cf. [12] and reviews in [13–15]), where they have often been manifest as predictive modeling that aims to use locational characteristics of known sites to establish criteria able to predict other probable site locations [14,16,17]. In addition to predictive modeling for practical research and management ends, locational analysis has also been used for analytical purposes, for example to characterize settlement locations and infer the preferences of the inhabitants. Here we focus on such characterizations, particularly with respect to the spatially-explicit and temporally-variable estimates of PAgP derived from the LPJmL agro-ecosystem model [10]. PAgP meets criteria recently argued to be necessary for relating climate changes to cultural trajectories [8,18,19]: it addresses human consequences, providing a derived variable that is climate-sensitive but also directly relevant to human experience.

Based on those analyses, we argue that climate-driven variability in PAgP, although clearly a reality for Holocene inhabitants of Provence, was of relatively modest magnitude–well within, we estimate, the reach of possible adaptive shifts in agricultural practice. Tolerance for declines and variability in agricultural production would thus have been primarily dependent on production targets and labor availability. Links between environmental changes and cultural changes would have been, as a consequence, contingent at least as much upon social/political/economic variables as environmental ones.

## 2. Holocene provence

### 2.1 The study area

This case study focuses on Provence, where variability in topography and geography produces marked bioclimatic diversity within short distances (cf. [20]) and which experiences significant interannual climatic variability. These contrasts make the development of methodologies able to capture fine-grained spatial and temporal variability vital for examining long-term human-environment interaction in the region. In addition, Provence has a richly documented archaeological record, much of which is available in digital form through *Patriarche*, as well as relatively high-resolution 20^th^-century climate data that can be used as the basis for downscaling paleoclimate reconstructions [11]. By focusing on an area that encompasses topographic diversity, abundant evidence of pre- and proto-historic settlement, and historically desirable and productive agricultural land, this case study addresses the consequences of Holocene climate change in the western Mediterranean at scales relevant to human inhabitants while taking into account the potential spatial diversity in the effects of climate change.

### 2.2 A brief cultural history

Before turning to empirical analyses, we contextualize our data with a brief overview of the broad archaeological patterns that can be described in Provence throughout the Holocene–i.e., a synthesis that aggregates across a topographically diverse landscape of >30,000 km^2^, from the Mediterranean to the headwaters of the Durance River in the Alps and from the Rhone into the southern Alps. While these patterns are not necessarily reflected precisely within the study area on which we focus here, the relationship of these broad patterns to the data for our study area suggests that our study area is, in general terms, representative of regional trends.

The date ranges in the discussion below reflect synthesis of research in Provence [21,22]. Although convention dictates use of dates in years BC by the Roman period if not earlier, we provide dates in years BP throughout to emphasize chronological continuity, and give dates in years BC in parentheses only where needed to orient the reader with respect to the cited literature. The *Patriarche* chronological assignments (in brackets) for sites within our study area, however, reflect the France-wide chronology employed in the *Patriarche* database, which does not precisely match an up-to-date chronology for Provence. Discussion of chronological issues, as well as additional detail on the archaeology of Holocene Provence, can be found in S1 Text.

Until the 8^th^ millennium BCE the inhabitants of Provence were exclusively Mesolithic–i.e., mobile foragers, with correspondingly modest population densities [23]. Throughout the Mesolithic and into the Early Neolithic (EN; approximately 7900–6750 BP), Provence was apparently relatively sparsely populated, albeit by increasing numbers of sedentary agriculturalists occupying small hamlets. The beginning of the Neolithic in Provence, probably some combination of in-migration by agriculturalists and adoption of agricultural technologies and lifeways by indigenous inhabitants [23–25], is marked by the first signs of widespread anthropogenic transformation of landscapes that until then had been little modified by human activity [26].

Neolithic agriculture focused on exploitation of cereals [27], primarily emmer wheat (*Triticum dicoccum*) [28], while einkorn (*Triticum aestivum*) and naked barley (*Hordeum vulgare nudum*) served as supplementary components. Archaeobotanical evidence suggests that Neolithic agriculture across a broad region of southern Europe comprised intensive small-scale farming [28–31], though there is still debate over the role of extensive slash-and-burn agriculture (see, for example, [32–34]).

In the Early Bronze Age (EBA; 4050–3550 BP [4250–3450 BP in *Patriarche*]), a reduction of human activity and population was accompanied by an overall increase in forest coverage [35]. Throughout the Bronze Age agriculture followed prior Neolithic traditions [36,37] and cereals remained dominant [38], but with a gradual diversification of crops and practices and significant innovations: the first evidence for animal traction [39], the introduction of additional cereals (broomcorn millet [*Panicum miliaceum*], and the beginnings of olive [*Olea europea*] cultivation in the region [40]). During the EBA and the Middle Bronze Age (MBA; 3550–3300 BP [3450–3150 in *Patriarche*]) the density of human occupation in Provence was at its lowest, following a notable decrease relative to previous phases. In contrast, the Late Bronze Age (LBA; 3300–2675 BP [3150–2700 in *Patriarche*]) is characterized by notable demographic growth and the emergence of new sites [21,41].

At the beginning of the Early Iron Age (EIA; 2675–2550 BP), a demographic decline is evidenced by the abandonment of many settlements. This was followed by the founding of the Greek colony of Marseille in 2550 BP, after which population growth is evidenced by both the foundation of new sites and the aggregation of population into larger sites [42]. However, this did not preclude a notable demographic decline around the year 2450 BP [43], just before the beginning of the Late Iron Age (LIA; 2400–2002 BP). Between 2071–2068 BP (121–118 BC), Provence became a Roman province (Transalpine Gaul). Several Celtic towns were abandoned at this time, but others continued to be occupied, and overall population density increased significantly, especially in the lowlands where agricultural exploitation increased.

In sum (see S1 Text for more detail), a period of several millennia of gradual increase in population size and density followed the introduction of cereal agriculture into the region, a trend that accelerated in the 5^th^ millennium BP. The demographics of the Bronze Age, roughly the subsequent millennium, were more variable, as population decline in the EBA was mirrored by population increase and settlement aggregation in the LBA. The subsequent abandonment of settlements in the EIA marks the beginning of a period for which we have more precise chronology of demographic change; from the beginning of the Iron Age onward archaeological chronologies based on material culture are sufficient to identify century-scale demographic changes (e.g., [44,45]), including demographic fluctuations following Greek colonization ca. 2550 BP (in the 6^th^ century BC) and significant population increase following Roman conquest ca. 2070 BP (towards the end of the 2^nd^ century BC). The landscape archaeology of Montagne Sainte-Victoire [45] provides a more local and detailed point of comparison, largely mirroring the regional pattern: comparatively minor Neolithic occupation is followed by a Bronze Age abandonment, after which settlement does not substantially increase until ca. 2150 BP (the end of the 3^rd^ century BC). The rich record for the later periods emphasizes that settlement decisions–foundation, abandonment, and location–took place within a complex web of social, political, and economic relationships. The relationships between site locations and PAgP that we describe here certainly did as well; the settlement patterns that we focus on below undoubtedly took form within social, political, and economic milieus. Nevertheless the strong patterning of settlement locations with respect to PAgP that we document below argues that agricultural productivity was a salient concern for the region’s inhabitants.

### 2.3 A Brief climate history

In the western Mediterranean at these latitudes, the most salient Holocene pattern is the establishment of Mediterranean vegetation–i.e. replacement of deciduous trees with evergreen sclerophyllous trees and shrubs–ca. 4500–4000 BP; this likely reflects some combination of climatic and anthropogenic forcings. Debate continues over the relative importance of these factors, with the shift in vegetation associated with the establishment of modern Mediterranean conditions alternately ascribed to general aridification or to impacts of expanding human populations and impacts (for recent reviews from both perspectives see, e.g., [46–49]). Centennial-scale variations in fluvial activity in the region are also argued to reflect climate variation (e.g., [50,51]), though imprecision in dating, regional variation, and issues of equifinality produce disagreement about the specific timings and causes of these episodes (cf. [51,52]) and the generalizability of particular local records across the region (cf. [53]). The overall picture is one of Holocene variability in the western Mediterranean, but diversity within the region in exactly how and when global or macro-regional patterns are manifest [54]. Magny and colleagues [55] have illustrated the complexity of climate change in the western Mediterranean by showing opposing aridification trends on either side of the 40°N parallel: the 4.2 ka event was the beginning of a dry period in southern Italy and the end of a dry period in France and Northern Italy. They relate that to larger-scale variation (e.g., the North Atlantic Oscillation (NAO)), while also noting that contrasts in seasonality may produce apparent contradictions in different proxy records.

This complexity extends to the local manifestations in the western Mediterranean of two climate “events”, at ca. 8200 BP and 4200 BP, that have been associated in the eastern Mediterranean with numerous arguments for climate impacts on human populations (e.g., [7,56]) (though some have questioned the focus on these periods at the expense of others (e.g., [4,57])). For instance, Magny and colleagues [58] identify the 4.2 ka event with a broader period of climatic oscillation from 4300–3800 BP in the central and western Mediterranean, Frigola and colleagues identify the same “event” from 4200–4000 BP in their Minorcan record, and it does not appear in the Alboran and Tyrrhenian Sea SST records [54]. The compilation undertaken by Weinelt and colleagues [59] is similarly ambiguous for the western Mediterranean.

### 2.4 Climate-Culture links

Arguments linking climate and culture change are much more common in the eastern than the western Mediterranean (e.g., [4,5,7,56]). This is likely due to some combination of the greater abundance of high-resolution paleoclimate records, larger and denser populations at times of significant Holocene climate change, and greater sensitivity to changes in precipitation in a generally more arid region. However, even in the eastern Mediterranean the causal links posited between climate and culture continue to generate significant debate. Fundamental critiques of the more speculative examples focus on the limitations of arguments that rest only on broad chronological correlations and claims of oversimplification, environmental determinism, and catastrophism, while even the more robust arguments have been subject to criticism about whether they sufficiently articulate specific links between climatic changes and cultural consequences, or are overly focused on “collapse” (cf. [60,61], inter alia).

This debate has been less salient in the western Mediterranean, where arguments for climatic influences on cultural phenomena are less common. Nevertheless, cultural trajectories have been linked to Holocene climate in the western Mediterranean in various ways, including both climate impacts on inhabitants and anthropogenic contributions to regional environment and climate; here we focus on the former. Berger [1] and Berger and Guilaine [2] argue that changes in subsistence and social patterns as well as the demographic increase associated with the Neolithic were linked to regional climate changes ca. 8200 BP. However, they point out also that a) any particular link between climate and the spread of Neolithic lifeways must be developed in detail for that case, keeping in mind the ways in which particular climates affected particular lifeways, b) the archaeological record itself may be biased by taphonomic effects associated with these climatic changes, and c) our chronologies for both climatic events and cultural trajectories remain relatively coarse, producing a potentially misleading abundance of correlations. In spite of these challenges, recent demographic reconstructions (e.g., [62, 63]) highlight correlations with climatic events at coarse spatial and temporal scales. Carozza and colleagues [3] more specifically link Mediterranean climate changes ca. 4200 BP to the EBA decline in population and anthropogenic landscape impact in southern France, citing particularly increased aridity and increased frequency of extreme precipitation events. In contrast, Weinelt and colleagues’ [59] attempt to link demographic changes at the beginning of the Bronze Age to climate changes ca. 4200 BP is, as they discuss, compromised by chronological imprecision and geographic variability; some of this difficulty may relate to the regional climatic complexity that Magny and colleagues [58] describe.

### 3. Data

**Fig 1:**
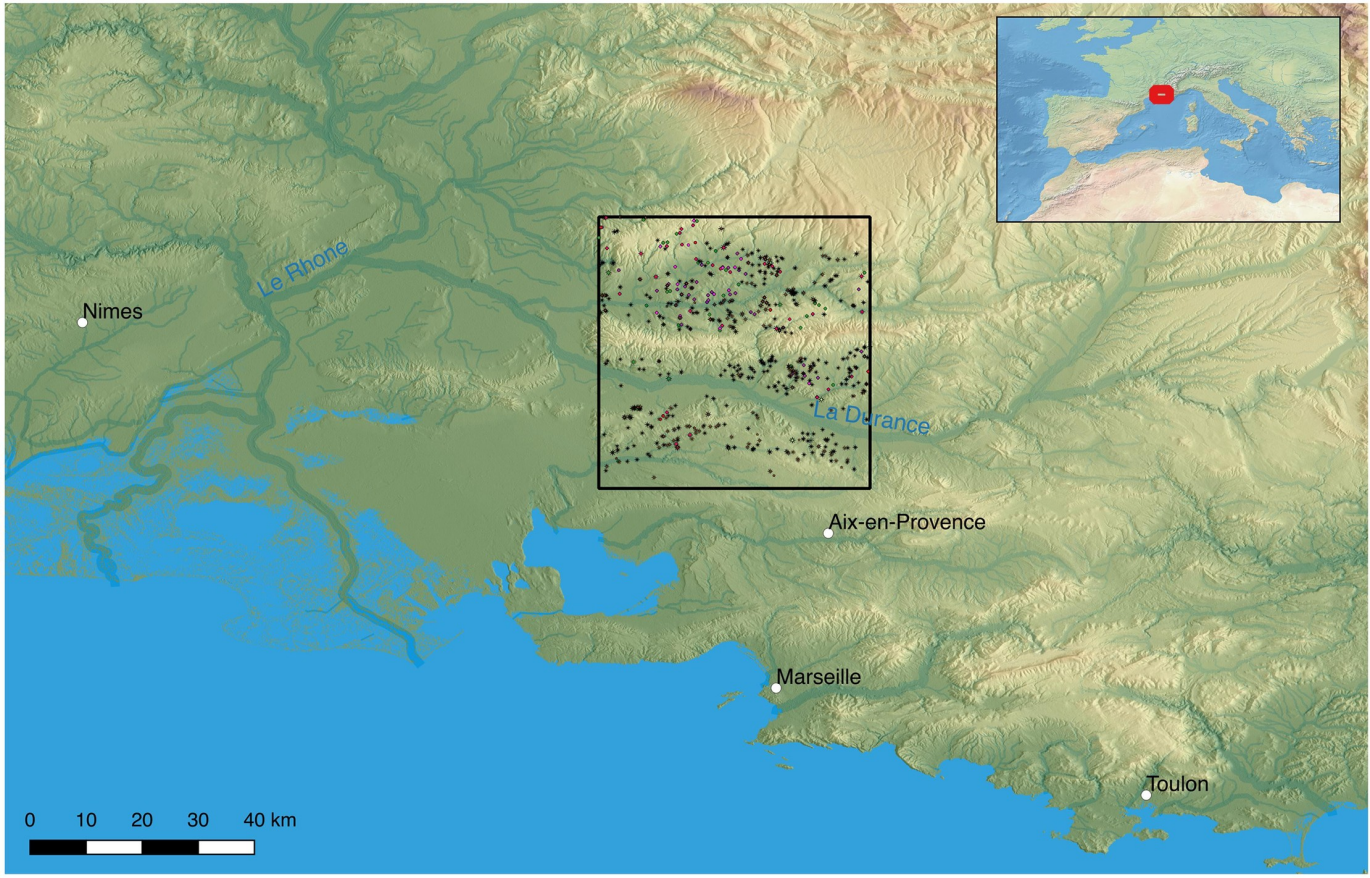
Study area and sites included in analyses (occupation and agricultural sites only, filtered to remove sites with uncertain chronology) from all periods (Early Neolithic through Gallo-Roman) from the *Patriarche* data. Note that where sites persist across multiple periods they overlap spatially, reducing the number of sites visible. Spatial data from NASA SRTM30 [64] and Natural Earth (http://www.naturalearthdata.com/).

We explore potential relationships between the inhabitants of our study area in Provence and climate changes through most of the Holocene (8400–1400 BP) by integrating empirical and modeled data. These include modern geographic data, climate-sensitive agro-ecosystem modeling of PAgP with annual resolution, and archaeological site data from the Early Neolithic through the Gallo-Roman period. For the study area of approximately 1400 km^2^ (Fig 1), we incorporate:

1. modern geographic data, primarily a 30m digital elevation model (SRTM30 [64]) and terrain characteristics derived from it,
2. high-resolution (300m pixels and annual steps) Holocene paleoclimate data produced by downscaling a 0.5° centennial-step modeled dataset derived from inverse modeling of pollen data for the Mediterranean throughout the Holocene ([65]; see [11] for methodological details),
3. annual 300m pixel results of the LPJmL agro-ecosystem model for the period 8400–1400 BP, parameterized for past agriculture through use of archaeological and ethnographic data (see [10]), produced using the high-resolution paleoclimate data described in (2) above, and
4. Archaeological site data spanning the period between 7250–1450 BP (the Early Neolithic period through the end of the Gallo-Roman period) from the *Patriarche* database, which provides coordinates, site classification, and chronological information for approximately 2250 sites (excluding those of uncertain date) in the study area.

**Fig 2:**
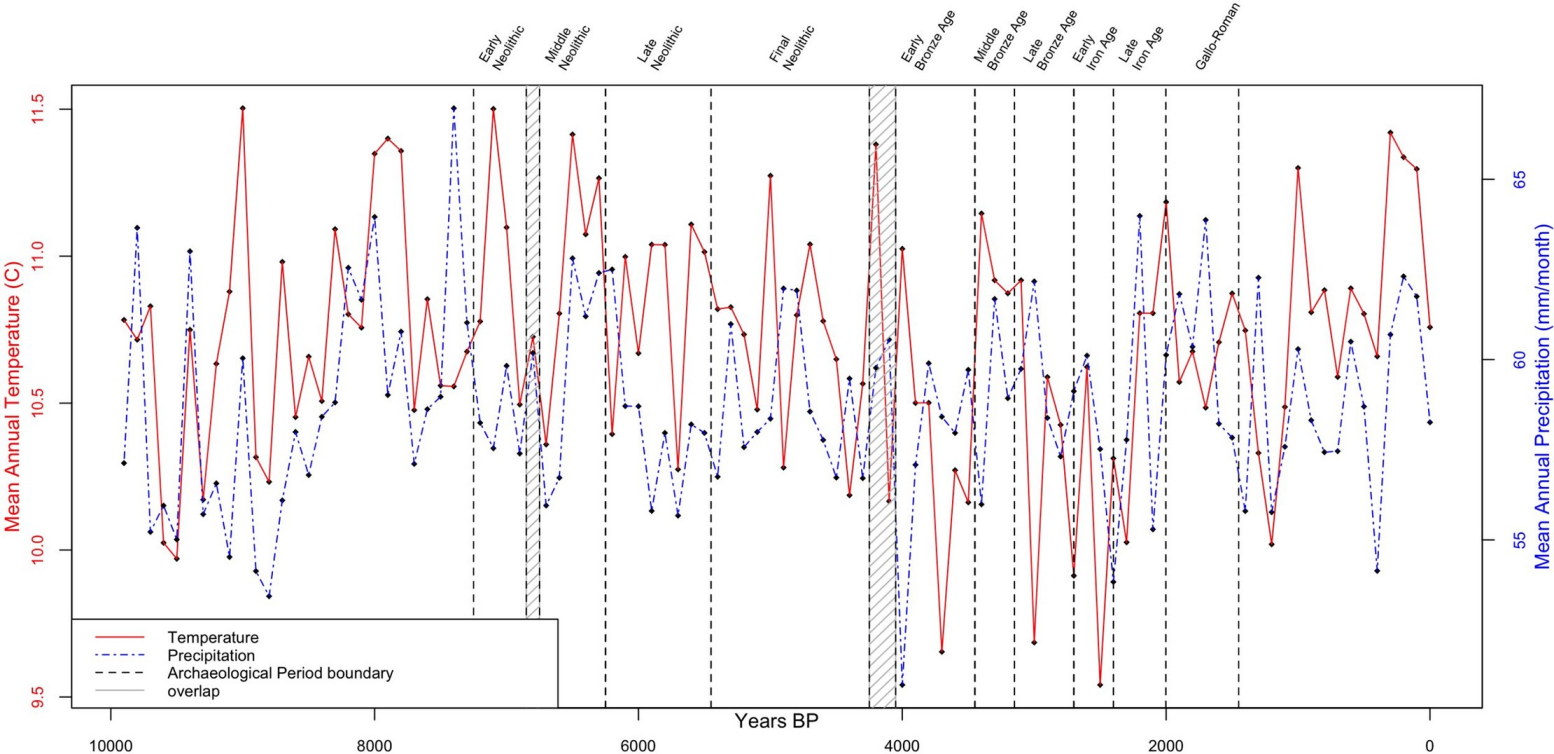
Holocene temperature and precipitation in the study area (centennial steps of annual means calculated from monthly values, from [65]), with cultural periods as defined in *Patriarche*. Data availability is detailed in S2 Text.

Centennial scale episodes of aridity are reflected in the subset of data from Guiot and Kaniewski’s Mediterranean-wide Holocene model [65] that we use here (see Fig 2). Although the accuracy and precision of the results might be improved by using more local proxy data, such data are unevenly distributed in space and time, generally lack the seasonal resolution produced by modeled data and necessary for agroecosystem modeling, and restrict the generalizability of the method. Employing Guiot and Kaniewski’s reconstructed climate dataset [65] makes it possible to easily replicate this method in any part of the Mediterranean Basin. Downscaling these data (cf. [11]) produces single probabilistic realizations based on the input paleoclimate data; individually these are realistic rather than accurate, but in aggregate they are as accurate as the input data allow, and enable re-aggregation across different intervals (e.g. in archaeological periods rather than centennial steps).

## 4. Methodology

Since a significant way in which climatic variables impact human inhabitants is through their effects on subsistence production (cf. [66]), we focus here primarily on the LPJmL results. LPJmL is a process-based agroecosystem model that builds on a dynamic natural vegetation model [67] to include agro-ecosystems [9,68], adding crop functional types (CFTs) to the established modeling process for natural plant functional types (PFTs). Using high-resolution and continuous paleoclimate data (see Section 3) and calibrating CFTs for past crops and agricultural practices allows us to produce spatially-explicit estimates of potential yields for cereals and pulses under various conditions of temperature and precipitation [10]. As archaeological evidence suggests that cereal crops were a more important staple in pre- and proto-historic Provence than pulses [36] and temperate cereal parameterizations have been the subject of greater development [9], we focus here on the results for wheat, taking that crop as broadly indicative of the productivities of other cereals. Moreover, given the relative scarcity of data about past agricultural practices and their diversity in time and space, we prefer to analyze a range of possible outcomes and therefore use two parameterizations, representing a low estimate (Wheat Par 2 [W2], approximating minimally intensive agriculture) and a high estimate (Wheat Par 1 [W1], approximating maximally intensive preindustrial agriculture, i.e. employing tilling, manuring, weeding, etc.) (for a detailed discussion of these parameterizations, see [10]). We focus primarily on contrasts between periods for a given parameterization, as these are the least dependent on necessary assumptions of the modeling process; discussion below is on the basis of W1 results unless otherwise specified.

After excluding sites of uncertain date and filtering the database of sites for occupational and agricultural sites–i.e., those most likely to be responsive to PAgP–we characterize the immediate catchment of each site by summarizing a series of variables for all pixels within a 200m circular buffer (i.e., a 12.6 ha area centered on the site; results for larger– 500m and 1000m –buffers are also reported in S1 Table, but we observed little variability contingent on buffer size and here discuss analyses only with the 200m buffer). These include both topographic variables and LPJmL results (see Table 1); we thus characterize both static variables and dynamic ones. In the case of the LPJmL results, the summary statistics for the space within each buffer are calculated from rasters that summarize each pixel value over time for the specified period; we examine both average values and variability. Although the modern data employed (the DEM and hydrologic network) are sub-optimal inasmuch as the modern landscape, particularly with respect to soil depth and vegetation cover, may be a far-from-perfect analog for the Middle Holocene one, retrodiction of past landscapes through inverse erosion modeling and paleovegetation reconstruction remains a future project. The R scripts designed for the analyses allow for future improvement in data sources and/or incorporation of additional variables.

**Table 1:** Variables summarized within each site buffer. W1/W2 and P1/P2 denote the high (1) and low (2) assumptions about agricultural intensity for wheat (W) and peas (P).

Variable	Data source
elevation (masl)	SRTM 30m DEM
slope (°)	SRTM 30m DEM
aspect (°)	SRTM 30m DEM
terrain ruggedness index (TRI)	SRTM 30m DEM
distance to fresh water (m)	SRTM 30m DEM + modern watercourses
soil texture (FAO classification)	ISRIC (https://soilgrids.org)
mean PAgP (tFM/ha)–W1/W2 and P1/P2	LPJmL (calculated per pixel from annual values spanning the relevant period)
σ PAgP–W1/W2 and P1/P2	LPJmL (calculated per pixel from annual values spanning the relevant period)

In order to assess the significance of PAgP (and changes therein) for inhabitants of the region in different periods, we employ both synchronic and diachronic analyses, comparing locations of distinct types of sites within periods and settlement locations across periods. PAgP of aggregated site catchments for each period is also compared to PAgP for the landscape as a whole during that period, contextualizing estimated land use within the universe of possibilities available to inhabitants. Further methodological detail and consideration of uncertainties is given in S3 Text and S4 Text.

Relating PAgP to settlement patterns echos previous archaeological attempts to relate settlement patterns to agricultural potential (e.g., [69, 70]), but with improved spatial and temporal resolution and detail; as Kvamme [13] points out, such fine-grained diachronic modeling of paleoenvironments can be critical to considering if, how, and when climate and environmental changes impact humans. In contrast to predictive modeling or much site location modeling, our goal is *not* to identify the predictors of site location, but rather to explore to what degree PAgP–itself variable over time and space–was an important factor (or not) in site establishment, persistence, and abandonment during different periods of pre- and proto-history. As discussed below (Section 5), this provides a means of empirically assessing the human consequences of climate dynamism.

### 4.1 Synchronic analyses

Synchronic analyses–i.e., investigation of sites from single archaeological periods–serve to check that we are not simply describing general changes in site location preferences that may covary with, but not be directly related to, PAgP. These analyses take two forms. First, where distinct categories of sites are located differently with respect to PAgP, and where the two groups vary independently from one another, we infer that the locations of settlement/agricultural sites are likely responding to particular rather than general imperatives. Second, we examine the distribution of sites against not only PAgP, but also against the other geographic variables that we have summarized within each buffer. Covariance amongst these (e.g., elevation and temperature; the latter influencing the LPJmL results) makes teasing apart their influences difficult, but where settlement distributions are more restricted with respect to one than to the other we argue that some selection is evident. Active selection may be more strongly argued still in the cases of newly established or abandoned settlements, though the smaller sample sizes of these categories limit their utility in some periods.

### 4.2 Diachronic analyses

**Fig 3:**
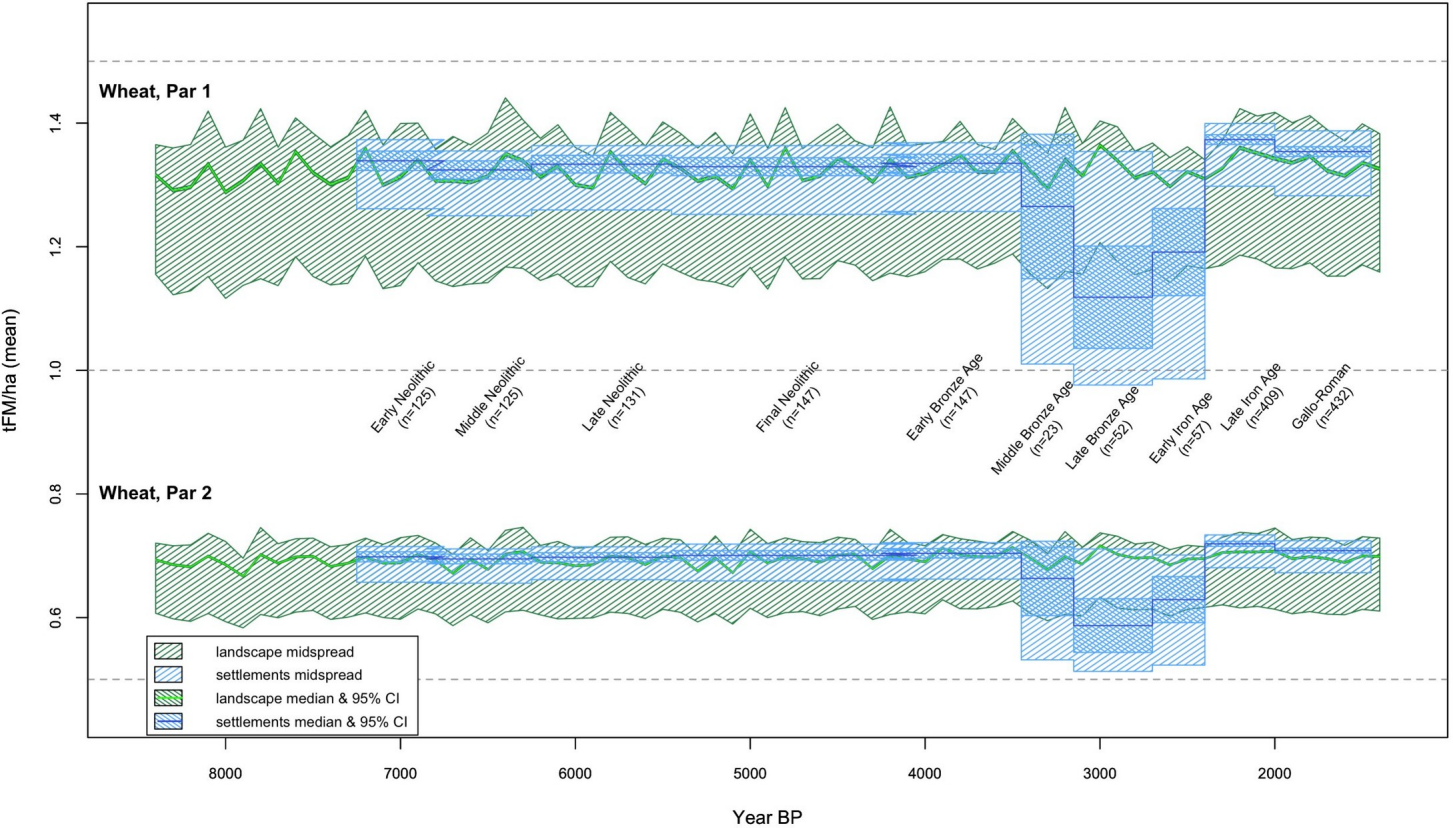
Potential cereal productivity of the landscape, in green, and of the exploited fraction, in blue; shaded areas are the midspreads, and the more darkly shaded areas the 95% confidence intervals around the medians. These are calculated with reference to all centennial mean pixel values (landscape) and the mean pixel values for each archaeological period within 200m buffers around each occupation/agricultural site (exploited fraction); see Section 5.2. The upper register is W1 (high agricultural intensity wheat) and the lower register, plotted on the same y-axis, is W2 (low agricultural intensity wheat).

Diachronic analyses focus on whether changes in settlement location between periods are correlated with changes in PAgP (absolute yields and variability in yields). Chronological resolution–period length–is again a limiting factor, as the averaging of PAgP across long periods minimizes variability (contrast the variability in temperature and precipitation in Fig 2 and the landscape PAgP in Fig 3 with the potential agricultural productivities of the archaeological periods in Fig 3). As a result we look also across period transitions. Although we lack the temporal resolution in the settlement data to examine associated changes in detail, we examine the severity of those events with regard to PAgP–i.e., how much would potential productivity have changed, how rapidly, and with what ubiquity?–and compare locations continuously occupied with those abandoned and those newly settled.

### 4.3 Managing uncertainties

The varying spatial and chronological resolutions of these datasets, as well as the quality of the data themselves, pose analytical challenges. Mismatches in spatial and temporal resolution in the geographic and paleoclimate datasets has been addressed through a downscaling approach [11] in order to produce the annual 300m pixel LPJmL results used here [10]. The resulting dataset is idea for analysis, but the accuracy of the downscaled data is limited by the accuracy of the regional-scale paleoclimate reconstruction that serves as the basis for the downscaling. In principle this could be improved by integrating local paleoclimate data for the study area with the regional reconstruction, but this presents another set of methodological challenges.

The *Patriarche* data present a different set of challenges, some endemic to archaeological survey data and others characteristic of data aggregated from diverse sources. These comprise primarily problems of chronological precision and landscape taphonomy, which raise questions of the size and representativeness of the samples of archaeological sites considered. These issues are discussed in detail in S4 Text.

Even a cursory glance at the spatial distribution of sites from all periods (see Fig 1) reveals a strikingly uneven site distribution: the ridge of the Luberon (running east-west across the center of the study area) and the floodplain of the Durance River (immediately south of the Luberon) are nearly devoid of sites, while with the exception of the northern and southern fringes all other areas are fairly densely covered. As with any archaeological site data–though in this case they are the result of synthesis rather than field survey–this pattern reflects the original site distribution, but is biased by differential site preservation and discovery. The two conspicuously empty areas are those of highest potential erosion (the Luberon) and deposition (the Durance floodplain) and thus suggest poor site preservation and discovery, respectively–though for these and other reasons they may also have been less appealing as settlement locations in the past.

Correcting for data biases without understanding those biases in more detail risks creating patterns out of nothing. However, considering the potential effects of taphonomy and time-averaging does demonstrate that the most salient patterns in the settlement data–the MBA decline in settlement and the LIA florescence–are robust with respect to likely biases. Applying the adjustment factors calculated by Berger ([71]; detailed in S4 Text)–not entirely geomorphically appropriate for our study area but arguably providing an appropriate worst-case scenario for taphonomic distortion–greatly inflates the number of EN and MN sites and suggests a marked decline in site numbers in the LN followed by modest growth in the FN; this dynamism within the Neolithic contrasts with the relative stability over time suggested by the raw data. Also in contrast with the raw data, the adjusted data (see rightmost columns in Table 2) suggest that heightened taphonomic effects on the EBA relative to the FN mask a significant increase in settlement in the EBA. At the same time, the effects of the time-averaging of sites across periods should somewhat counterbalance the emphasis that the adjusted data put on the Neolithic Period, as longer periods are likely to have the effect of overemphasizing numbers of sites (as is evident in the time-adjusted counts in Table 2). In short, the patterns we consider below appear to be robust even in face of a worst-case assumption about biases in the data.

**Table 2:** Site counts by period (sites may persist for multiple periods so column totals may be higher than the total number of sites recorded in the region). All counts follow filtering to exclude sites of uncertain chronological affiliation. The time-adjusted counts are calculated by dividing the total number of occupation/agricultural sites by the period length standardized to the shortest time period (e.g., for the EN, 125/(500/300)), following the logic detailed in [72]. The three rightmost columns are site counts adjusted following Berger’s findings in the Middle Rhone Valley [71].

Period	Total number of sites	Number of occupation/agricultural sites	Number of newly-established occupation/agricultural sites	Number of occupation/agricultural sites abandoned since previous period	Period length (years)	Time-adjusted counts (sites / standardized time period)	Berger adjustment factor [71]	Adjusted estimated number of sites	Adjusted number of occupation/agricultural sites
**Early Neolithic (7250–6750 BP)**	144	125	125	0	500	75	47.18	6794	5898
**Middle Neolithic (6850–6250 BP)**	144	125	0	0	600	63	27.5	3960	3438
**Late Neolithic (6250–5450 BP)**	151	131	7	1	800	49	15.97	2443	2092
**Final Neolithic (5450–4050 BP)**	181	147	22	6	1400	32	15.97	2923	2348
**Early Bronze Age (4250–3450 BP)**	177	147	0	0	800	55	25.27	4523	3715
**Middle Bronze Age (3450–3150 BP)**	36	23	0	124	300	23	25.27	960	581
**Late Bronze Age (3150–2700 BP)**	82	52	32	3	450	35	25.27	2123	1314
**Early Iron Age (2700–2400 BP)**	87	57	5	0	300	57	9.7	863	553
**Late Iron Age (2400–2002 BP)**	574	409	365	13	398	308	9.7	5849	3967
**Gallo-Roman (2002–1450 BP)**	610	432	24	1	552	235	8.65	5553	3737

Drawbacks of the dataset are offset by the remarkable density and time depth of the data. Moreover, the most salient bias in the data–i.e., the absence of data from the Durance floodplain–is consistent over time, suggesting that diachronic analyses should be minimally affected. Comparative spatial analyses are more vulnerable, but by focusing on presence data rather than absence data we attempt to minimize this problem. That is, we emphasize characterization of landscape areas where sites *are* recorded, rather than taking absence of sites as evidence of absence of occupation. Where sample sizes allow we look also at establishment and abandonment of sites from one period to the next.

## 5. Results

### 5.1 Diachronic settlement patterns

Interpreting changes in the number of sites over time is complicated by landscape taphonomy and time-averaging (see Section 4.3), and as data about site size is inconsistently reported we exclude that also. Nevertheless some patterning is strong enough that it likely transcends problems of site preservation or chronological limitations, and the presence of sites from all periods in most areas suggests that taphonomic problems are at least not effacing particular periods from the landscape. Within the study area, the most salient diachronic pattern is a marked MBA abandonment; resettlement of the area began in the LBA and a florescence of sites followed in the LIA and continued into the Gallo-Roman Period.

These patterns are broadly coherent with generalized archaeological understandings of long-term occupation history of Provence (see Section 2), though they do not conform exactly. While the general outline of intensifying occupation in the later Neolithic followed by a Bronze Age decline is certainly apparent locally, the decline is pronounced, in the local data, in the MBA rather than the EBA as suggested regionally (though given that these abandonments have only a *terminus ante quem* of the end of the EBA, this apparent offset from the regional pattern may also in part be an artifact of data resolution). The regional population decline in the EIA is reflected locally in the slower pace of new settlement relative to the LBA. Local chronology (i.e. the periodization used in the *Patriarche* dataset) is not sufficient to resolve regionally-apparent fluctuations during the LIA and Gallo-Roman periods [44]; as a result the apparent burst of settlement in the study area at the beginning of the LIA is likely the result of overall growth throughout that period. At the same time, the rearrangement in settlement patterns during the LIA–i.e., abandonment by the LIA of some EIA sites even as the total number of settlements increased dramatically–is consistent with the regional political upheaval of the time.

The robustness of some of these patterns argues that they are not simply artifacts of the known biases in the data, but rather reflect archaeological patterning produced by changes in the scale and density of human habitation. These changing settlement patterns within the study area are those that we query regarding the role of changes in PAgP.

### 5.2 Assessing the SIGNIFICANCE of PAgP

Settlement locations reflect the significance of PAgP imperfectly, as changes in location (or persistence of existing settlements) may respond also to other imperatives, e.g., political realities, economic relationships, landscapes of social and symbolic significance, demographic changes, etc. Part of the responsiveness of any settlement pattern to changes in PAgP obviously depends also on the relative importance of agriculture, and particularly of agriculture targeting high yields. That is, if modest yields are sufficient, or agriculture a minor component of the economy, then changes in potential productivity may have less impact. The significance of changes in potential agricultural yields that we examine here would have depended also on how significant agriculture was for inhabitants; in Provence agriculture has been since its origins mixed, in varying proportions, with foraging and pastoralism.

**Fig 4:**
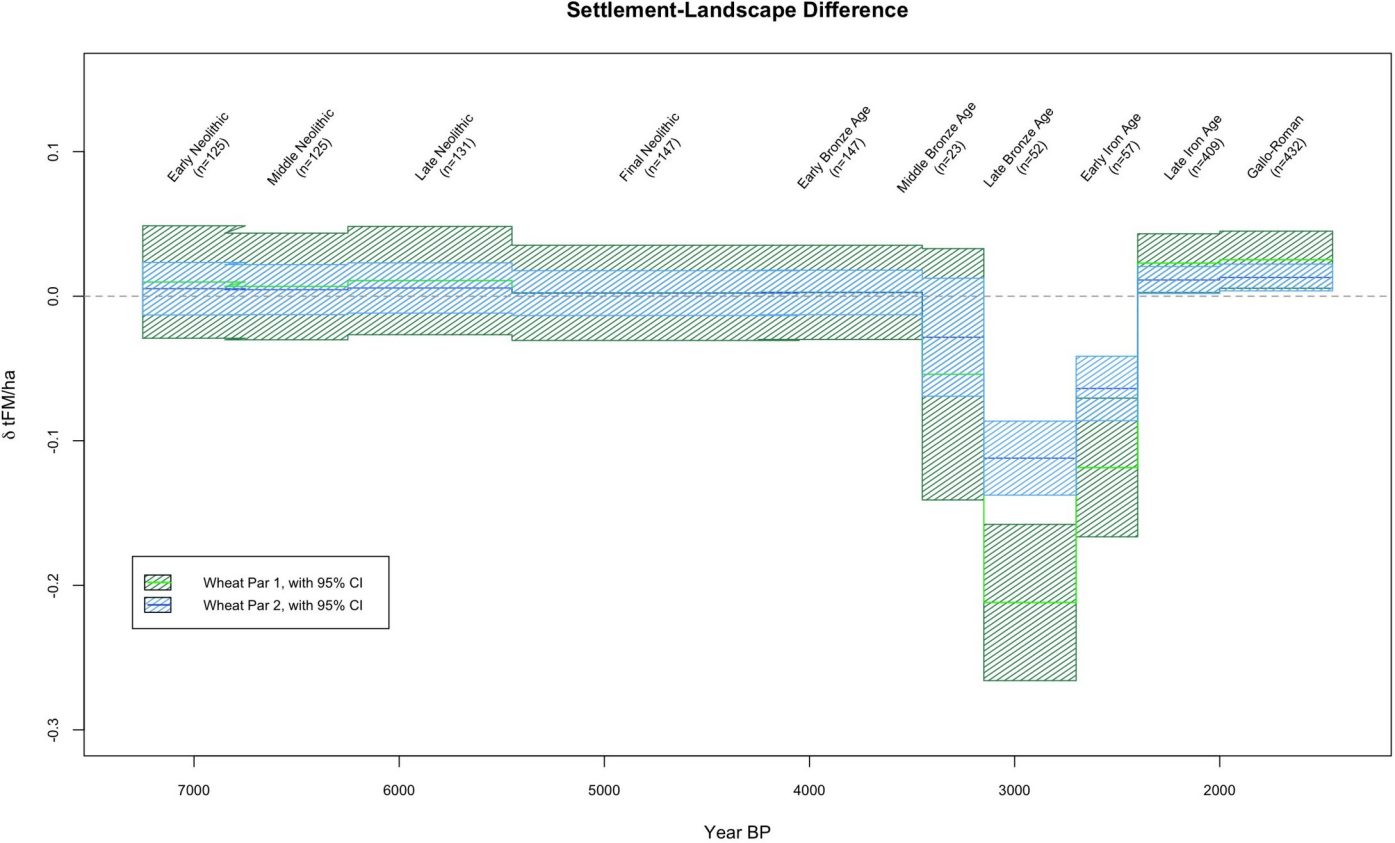
Difference between the exploited fraction of the landscape and the landscape as a whole (note that the abrupt transitions between periods are a function of the periodization).

As discussed in Section 4, a vital challenge in locational analysis with respect to environmental variables is the need to contrast locations with the landscape as a whole–that is, the array of *possible* settlement locations. We illustrate this here (in Figs 3 and 4, and S2 Fig) using the median and midspread landscape values calculated from the mean PAgP (tonnes of fresh matter per hectare [tFM/ha]) values per pixel, averaged per century (or in some cases per archaeological period). This produces, for a given timespan, a single raster containing one summary value for each pixel (generally the mean, though we have considered also standard deviations in the analyses presented here). The median and midspread of that raster (i.e., summaries of the mean pixel values over time across the landscape) then provide a summary of the potential productivity of the study area for a given period. These can be compared to the median and the midspread of the fraction of the landscape exploited by the inhabitants, characterized by summarizing the statistics calculated over the 200m buffer around each site (see Section 4).

The aggregated data for each period demonstrate that occupation and agricultural sites occupy cells that are not a random sample of the study area: the midspread of the PAgP values for the exploited fraction of the landscape is (with the exception a few anomalous periods, discussed below) centered at or above the landscape median, and is restricted in its range to the upper part of the landscape midspread (Fig 3). While this likely reflects at least in part general avoidance of high altitudes and steep slopes, which are negatively correlated with potential productivity, it is also clear that these agricultural and domestic sites–site types whose location was most likely tied to subsistence decisions–occupy a more restricted range of the landscape than other sites (see S1 Fig). These locations can be argued, then, to reflect not simply generalized settlement patterns but particular attention to PAgP (or, potentially, to other variables that correlate with it).

### 5.3 Settlement patterns and PAgP over time

**Fig 5:**
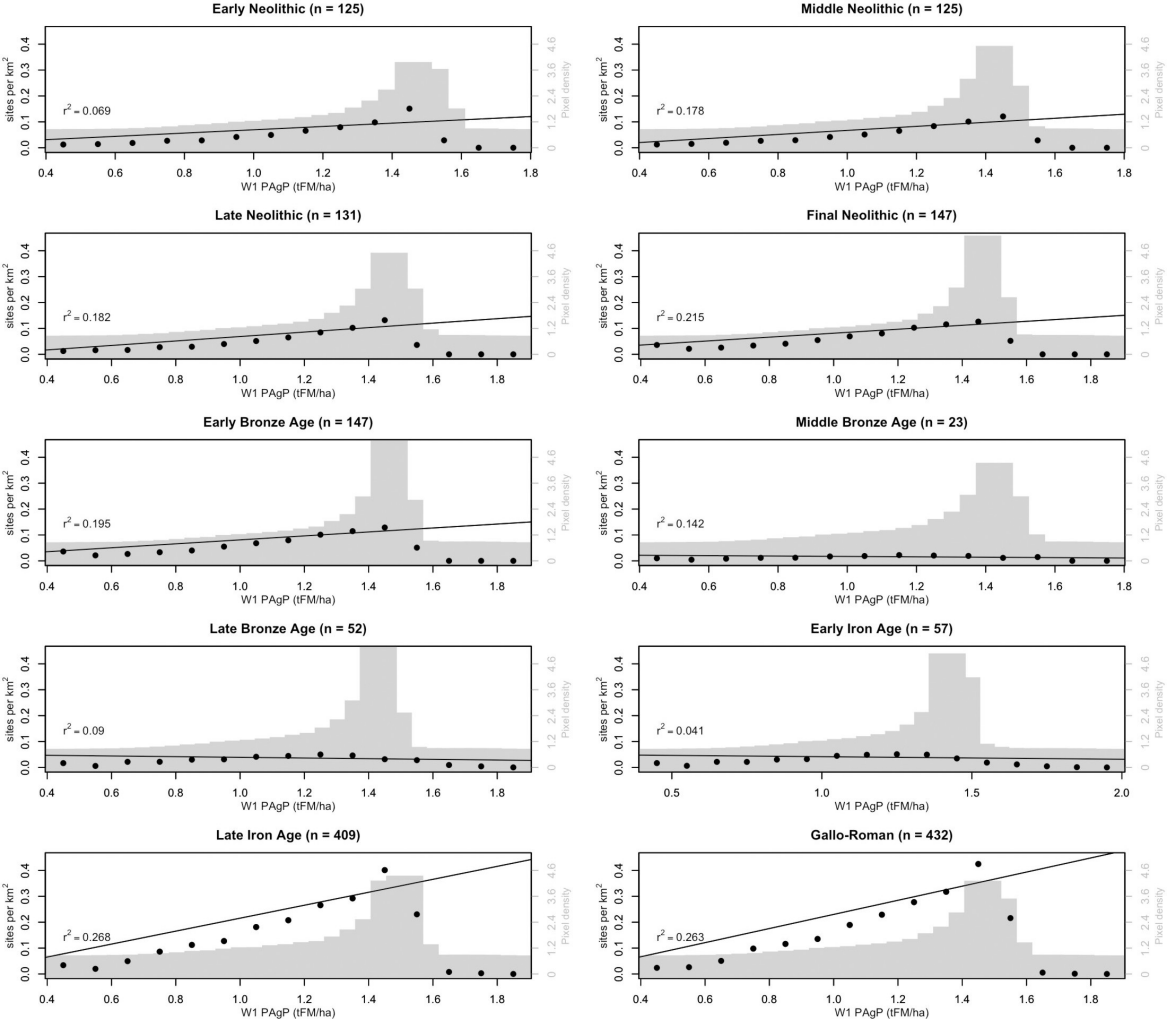
Univariate regression of site intensity (sites/km^2^) against binned PAgP values for each archaeological period. PAgP is binned in increments of 0.1 tFM/ha.

Throughout the Neolithic and into the EBA, the portion of the landscape exploited by inhabitants comprised a fraction of the available landscape with higher-than-background PAgP values (see Figs 3 and 4). A radical departure from this pattern followed in the MBA, at which point the PAgP values of the exploited fraction of the landscape can be seen to be almost entirely below the median landscape value. This pattern is even more accentuated in the LBA, and persists into the EIA, before another dramatic shift accompanied the demographic expansion of the LIA, when settlements again began to occupy areas of higher PAgP than the landscape median. These changes in settlement pattern are associated with the broad depopulation of the area in the MBA, which considerably reduces the sample size (number of settlements) for used to calculate the exploited fraction of the landscape during the MBA-LBA-EIA relative to all other periods (reflected in the loosening of the confidence intervals in Figs 3 and 4). However, the pattern is strong enough that only in the MBA is there any ambiguity about whether the PAgP of the exploited fraction is significantly different than the landscape background (see Figs 3 and 4 and S2 Table). Although causal relationships are difficult to infer due to the covaration between PAgP and geographic variables like elevation and slope, the correlation between settlement intensity (sites/km^2^) and PAgP values can be clearly seen to vary over time in a univariate regression of the former with binned values of the latter (Fig 5, following the method outlined in [73]).

The changes in the PAgP of the exploited fraction of the landscape from the EN through the EBA track the climate-driven changes in the potential productivity of the landscape as a whole fairly closely (Fig 3 illustrates the changes in landscape PAgP over time, while Fig 4 illustrates the difference between exploited fraction and landscape over time, aggregating all data to the chronological resolution of the *Patriarche* data). While the PAgP of the exploited areas varies from the EN through the EBA, that variability is a function of changes in the environment rather than changes in site location (while site locations may change, they do not systematically shift towards areas of higher or lower PAgP). In contrast, throughout the MBA, LBA, and EIA, the inter-period differences in the fraction of the landscape exploited appear independent of the background environmental change. Both settlement locations in these periods, and the changes between them, argue that during these periods PAgP ceased to have any structuring influence on settlement location. In contrast, that relationship appears to have become even more salient in the LIA and G-R periods, when the median value of the exploited fraction of the landscape again climbs above the landscape background, reaching its highest levels relative to the landscape median (Figs 3 and 4) and when correlation between settlement intensity and PAgP is strongest (Fig 5).

These changing relationships to PAgP over time create stark contrasts between the EBA and MBA, when the number of settlements drops precipitously and they begin to occupy areas of significantly lower PAgP, and the EIA and LIA, when the number of settlements climbs dramatically and their locations appear more concerned with PAgP than ever before. As we have discussed in Section 3, the coarse periodization of the archaeological data makes these contrasts between periods seem more abrupt than they likely were, but nonetheless the contrasts are salient.

#### 5.3.1 The MBA-LBA-EIA anomaly

**Fig 6:**
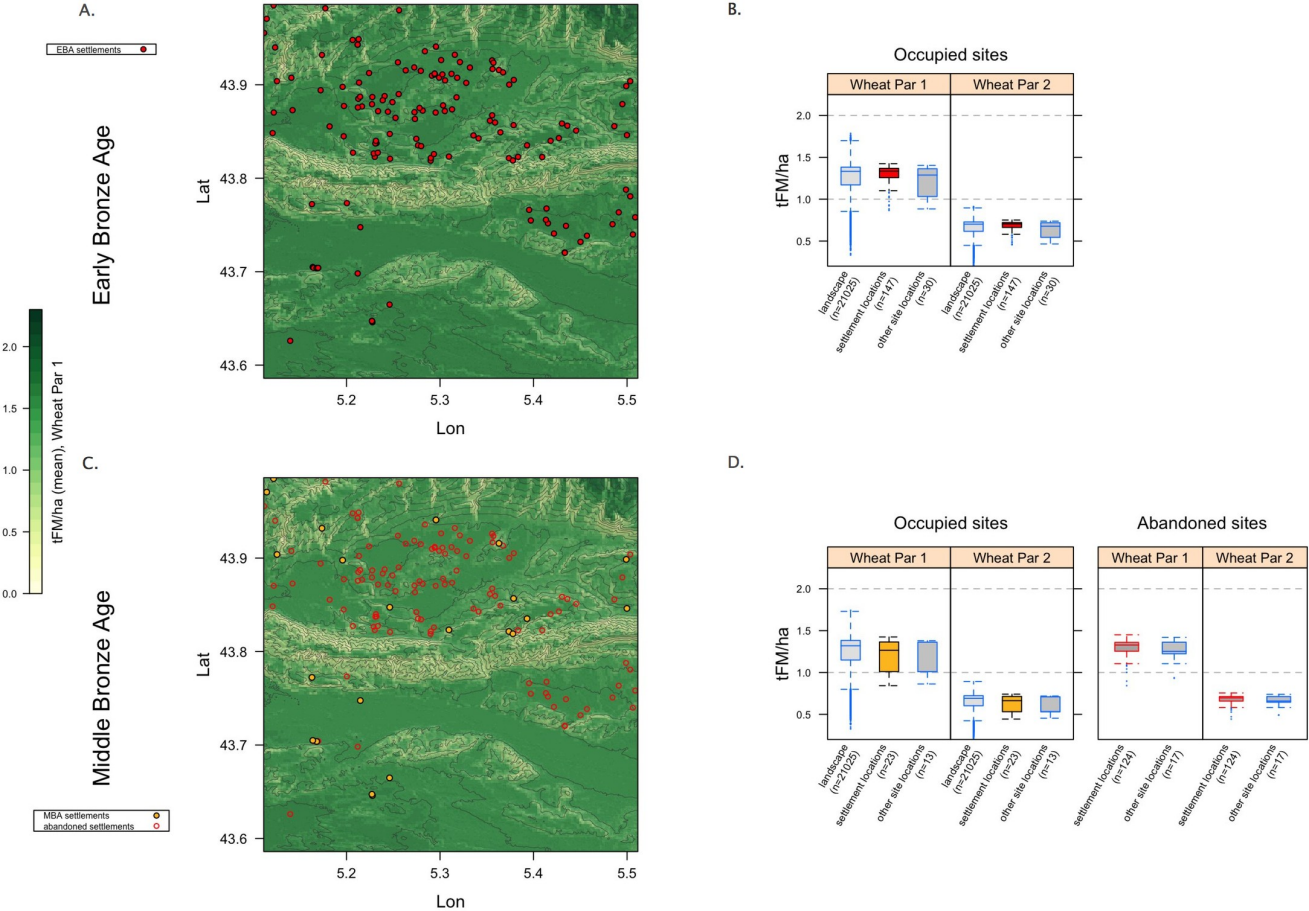
EBA (a) and MBA (c) settlement patterns; the latter includes also sites that had been occupied in the EBA but were abandoned by the MBA. The boxplots (b and d) aggregate the PAgP values of the landscape as a whole as well as the exploited fraction and that occupied by other types of sites; (d) also includes those data for the abandoned sites, demonstrating that the sites that were abandoned were associated with land with high PAgP values. The raster background shows W2 (low agricultural intensity wheat) values but both W1 and W2 are included in the boxplots and the pattern is robust across the two.

Fig 6 plots EBA and MBA settlements with respect to PAgP; Fig 6C and 6D include not only MBA settlements but also those sites that had been occupied in the previous period. The abandonment was substantial, and the boxplots (Fig 6D) highlight that the sites that were abandoned were associated with land of *higher* PAgP. MBA occupation/agricultural sites were located in areas with a broad spread of PAgP values, but below the landscape median and similar to sites of other types (Fig 6D). While the small sample size for the MBA makes the significance of that contrast somewhat ambiguous, the same pattern for the LBA and EIA is strong enough to be significant in spite of the relatively small sample size (S2 Table). This pattern (see Figs 3 and 4) is robust across both high and low scenarios of agricultural intensity (i.e. W1 and W2). It is also evident in our modeling of pulses, for which landscape PAgP is more variable than for cereals, but for which PAgP of the exploited fraction exhibits an analogous MBA-LBA-EIA anomaly (S2 Fig).

PAgP was clearly *not* a primary concern for some considerable lapse of time between the end of the EBA and the beginning of the LIA, suggesting that a) other imperatives were *more* important (that this shift was to higher and steeper ground–see S1 Fig–suggests perhaps a concern with defensibility), and/or b) alternative (non-agricultural) subsistence resources may have increased in importance. The shift in habitation from the most potentially agriculturally productive parts of the landscape to significantly poorer areas represents, we would argue, a shift from situations where agricultural productivity was an important influence on site location to periods when other imperatives overwhelmed agricultural production. The uncertainties during these periods increase as the sample size of sites shrinks dramatically, but the pattern is so strong that it transcends these uncertainties, and in fact the reduction in sample size is itself a significant aspect of the diachronic settlement pattern. People were apparently abandoning the region, while those that remained shifted to much less productive areas. Not until the LIA was this pattern reversed and highly productive land occupied again.

#### 5.3.2 The late iron age and the gallo-roman period

**Fig 7:**
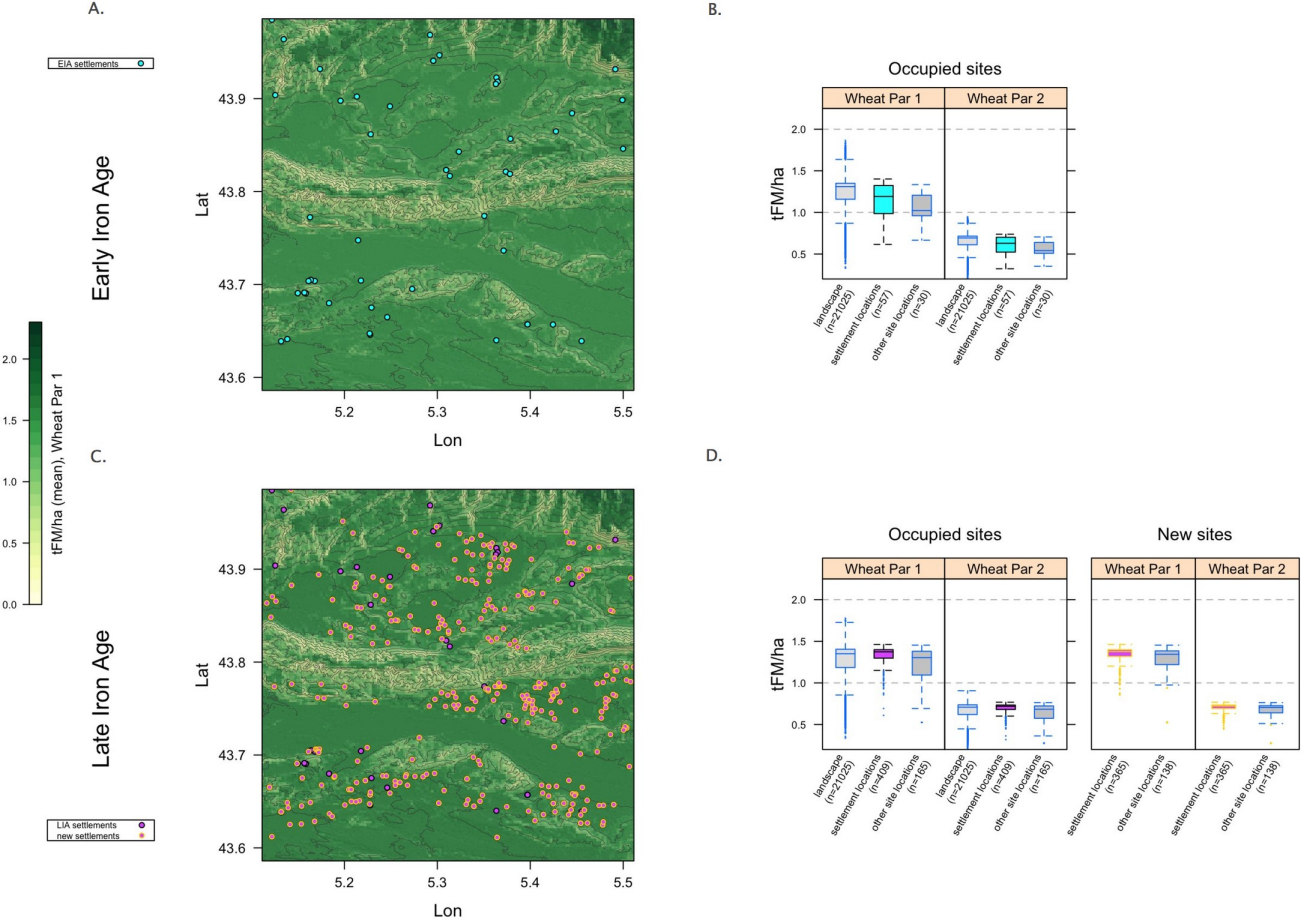
EIA (a) and LIA (c) settlement patterns; the latter highlights sites newly established in the LIA. The boxplots (b and d) aggregate the PAgP values of the landscape as a whole as well as the exploited fraction and that occupied by other types of sites; (d) also includes those data for the new sites, demonstrating that new sites were established on land with particularly high PAgP values. The raster background shows W1 (high agricultural intensity wheat) values but both W1 and W2 are included in the boxplots and the pattern is robust across the two. As is evident in S1 Fig, this pattern also involved a resettlement of lower-elevation areas.

The intensification of settlement in the LIA (Fig 7C) is characterized not just by a dramatic increase in the number and density of sites, but also by populations that appear to have had much more interest in the potential agricultural productivity of the areas they occupy (or perhaps freedom to occupy those areas). Where in the EIA sites were located without apparent concern for PAgP (that is, the median of the exploited fraction is below the landscape median, the midspread is broad, and occupation/agricultural sites are relatively similar to other sites; see Fig 7B), in the LIA the newly established occupation/agricultural sites occupy areas with a narrow range of high PAgP values, in marked contrast to other site types (Fig 7D). In the LIA and the GR periods, moreover, the median PAgP of the exploited fraction of the landscape is higher above the landscape median than at any previous point (Fig 4), even though the landscape itself was in a climate-driven period of maximal Holocene PAgP.

## 6. Discussion

The conspicuous deviation from the long-term pattern is the MBA-LBA-EIA period. During that span the divergence from the long-term tendency to exploit areas of high PAgP relative to the landscape background is salient enough to be significant in spite of the small sample sizes of the period, and is not apparently attributable to taphonomic or sampling biases. It is notable that this shift during the MBA-LBA-EIA is relative as well as absolute: the PAgP values of exploited areas drop even while background landscape PAgP remains relatively high (see Figs 3 and 4). This argues that the MBA-LBA-EIA pattern is not attributable to climate-driven environmental deterioration, and in fact the nadir of exploited PAgP values in the LBA occurs during a period of increased background PAgP relative to the periods immediately before and after (Figs 3 and 4).

Similarly, the LIA-GR increase does not appear to be attributable to climate-driven increase in the PAgP of the landscape as a whole. Although landscape PAgP does increase at the beginning of the LIA, reaching some of the highest levels of the Holocene by 2200 BP, the exploited fraction values for these periods are not only high but also high relative to the landscape background. The high PAgP values associated with LIA and GR settlements are evidence that sites in these periods were, even in a landscape of markedly high PAgP, nevertheless exploiting particularly productive areas.

These apparent decouplings of changes in settlement patterns from climate-driven changes do not rule out the possible impact of some fairly rapid changes in potential agricultural productivity. For instance, although the changes in settlement pattern from the FN to the EBA are modest, the transition between periods is coincident with a dramatic decline in PAgP (-10% in the landscape median between 4200 and 4000 BP), and the number of site abandonments *during* the EBA is significantly higher than in any other period (though these abandonments may well have occurred centuries after 4000 BP). Conversely, the LIA, the period of the single largest increase in settlement, coincides roughly with the most marked increase in PAgP of the Holocene (+11.5% in the landscape median from 2400–2200 BP).

Without archaeological data of higher chronological resolution, however, any attempts to link diachronic settlement patterns to climatically-driven variation in PAgP remain speculative at best. The limitations of the archaeological chronology make correlating any of the more dramatic inflections in landscape PAgP (Fig 3) with change in settlement pattern impossible, while the contrasts between long-term PAgP averages (Fig 4) are of relatively small magnitudes, making them unlikely candidates as drivers of change. Moreover, even the more dramatic changes in median landscape PAgP (over the spans 4200–4000 BP and 2500–2200 BP) are of magnitudes ranging from 6–11.5% (see Fig 3 and S2 Table). These magnitudes are roughly six times lower than the changes in cereal yields potentially achievable through changes in agricultural intensity (i.e., the space between the two registers in Fig 3).

The fairly modest scale of climate-driven changes in productivity relative to potential human-managed changes in productivity does not constitute an argument that inhabitants would have been impervious to environmental changes. Rather, variation in potential productivity is of magnitudes suggesting that societal impacts would have depended on such variables as target production, ability to shift locations, practices, and/or crops, labor availability, non-agricultural subsistence alternatives, and participation in networks of trade and exchange. Many forms of agricultural intensification (in addition to extensification and relocation), ranging from simple labor inputs (e.g., increased tilling and/or manuring) to complex landscape modification (e.g., construction of terracing or raised fields) were practiced by preindustrial agriculturalists globally (cf. [74]). Thus, while Fig 3 illustrates that there were changes in PAgP over time, whether those changes were significant for inhabitants of the area depends on many additional factors.

While a reduction on the order of 10% might not be catastrophic unless production needs/targets/desires were near the top of those achievable in W1, nevertheless there would have been social consequences of such adaptation. An increase in labor input sufficient to make up a 10% reduction in yields (by, for example, increasing manuring, tilling, and weeding, or by extensification of cultivation) would have social/political/economic ramifications even if it were practicable. Those ramifications would have varied depending at least as much on societal variables (demographic, political and economic factors driving production demands, labor availability and organization, etc.) as on the magnitude and rate of any change in climate. For instance, the large potential for improvement in yields from a W2 baseline via changes in agricultural practices, and the relative ease of shifts in location and extent of farming at low population densities, are consistent with the high resilience that Flohr and colleagues [75] argue are characteristic of early farming societies in Southwest Asia. Such resilience may also be related to the relative dampening of variability in W2 as compared to W1 (compare the two registers in Fig 3).

Impacts of climate changes–at least, those impacts manifest as and through changes in agricultural yields–would have arisen in limited circumstances, primarily determined by production imperatives. If production needs/targets were near the top of W1, any climatically-induced decline in PAgP would have had impacts that could not have been offset by changes in agricultural practice. In cases in which production had to be maintained but was not near the upper limit of W1, however, intensifying and/or extensifying agriculture could have maintained yields, provided that increasing labor inputs, adopting different technology, and/or shifting the locations of agricultural production were viable options. Subsistence would not have been imperiled except at levels below ~1.0 tFM/ha (a rough threshold based on Halstead’s [76] ethnographic work in 20^th^ century Greece on what cereal yields could sustain subsistence farmers), never affecting more than a fraction of the study area landscape even under W2. It bears re-emphasizing, however, that these values are long-term means; a different metric would be necessary to get at the frequency and/or severity of short-term fluctuations, and the impacts of short-term fluctuations in PAgP would be influenced by social/political/economic factors in distinct ways. It is also important to note in this context that the scale and structure of interannual climate variability is derived from 20^th^-21^st^ century climate variability through downscaling with a mechanistic model and conditionally stochastic methods [11]. This limitation in available data limits confidence in the accuracy of past variability.

Assessing the consequences of variability for inhabitants remains as much a theoretical problem of which aspect(s) of variability in production are significant as a methodological problem of modeling variability. We have focused here on mean PAgP/pixel over time, but one of the interesting questions raised by examining long-term variation in PAgP/pixel is that of which metrics of agricultural yield over time were relevant to past inhabitants, and whether those did change over time (e.g., absolute annual yields, annual yields relative to other areas, interannual variability in yields, frequency of yields low enough to be undesirable, etc).

This is related to the problem of time-averaging: as we have mentioned (Sections 4.2 and 4.3 and in S4 Text), variability in exploited areas is downplayed by the time-averaging imposed by long time periods, limiting our ability to detect even simple correlations between changes in settlement patterns and particular climate changes, much less impacts of short-term fluctuations. This is a problem only really solvable with archaeological data of finer temporal resolution. Improving data centralization (e.g., ArkeoGIS [77,78]) promises to help, but remains limited by the chronologies of input data (and, in data aggregation efforts, by the lowest common denominator, i.e. the data with the poorest chronological resolution; synchronicity across regions can also be an issue [see discussion of the challenges of employing the *Patriarche* chronology in Section 2.2 and in S4 Text]). Improving site and regional chronologies through Bayesian modeling should eventually improve the achievable chronological resolution considerably [79–81], but will require revisiting of archived data if its potential is to be realized with regional datasets.

## 7. Conclusions

The relative magnitudes of a) climate-induced variability in PAgP, and b) variability in PAgP contingent on agricultural practices suggest that:

1. except in cases when target/desired/required yields were near the upper limits of W1, Holocene climate shifts were of magnitudes whose impacts could potentially have been offset by local populations through changes in agricultural practices (primarily involving increases in labor inputs, e.g., increased manuring, tilling, and weeding, and/or extensification or relocation of cultivated areas), and
2. whether sufficient compensatory responses could be adopted, and what the ramifications of those responses were, would have been a function of sociopolitical factors (e.g., availability of labor and land, desired or required yields, and ability to weather shortages through storage, trade and exchange, mobility, and regional networks, etc.).

Vulnerability thus has to be discussed contextually, with reference to particular societal conditions, and cannot be characterized with reference to simple climatic thresholds (with the possible exceptions of situations in which production demands are near the upper possible limits, or in marginal environments in which intensification or extensification cannot overcome environmental limiting factors). As vulnerability and resilience are contingent on *both* characteristics of climate changes *and* characteristics of society and economy, arguments relating climatic and societal changes have to be grounded in analysis of the specific human consequences of particular environmental changes. That is, such arguments must explore, as we have begun to here, the mechanisms through which climate change might have affected inhabitants.

Improvements in input data can of course improve the accuracy of the results, and modeling might be expanded to include a) more crops with potentially different relationships to climate variables and resultant different geographic patterning of potential productivity, and b) feedbacks linking human activity and changes in the environment that impact potential productivity (e.g., erosion resulting from land-use). LPJmL (or other crop models) can include additional crops (cf. [9,68]) and could be developed to more specifically represent pre-industrial agricultural practices, while spatially-explicit erosion modeling might also be incorporated (e.g., [82–85]). Linking these elements in a complex socioecological systems model to explore the variables conditioning vulnerability and resilience remains an important research goal [18,86], and has been productively explored in other regions through such tools as agent-based modeling (e.g., [87–90]).

Even with the limited modeling that we have employed here, and with the limitations in chronological resolution described in Section 4.3, we have been able to demonstrate that inhabitants of Holocene Provence were primarily exploiting the parts of the landscape with higher potential agricultural productivity. The notable exception to this pattern is the marked shift spanning the MBA-LBA-EIA, when other imperatives apparently drove inhabitants to less productive areas.

This case study demonstrates that downscaled paleoclimate data in conjunction with agroecosystem modeling has the potential to shed light on the human consequences of climatic changes. The spatially-explicit and diachronic calculation of a climate-sensitive variable that directly measures an impact on human communities of climate dynamism constitutes a mechanism that can link climate variability with cultural change–or, as in the MBA-LBA-EIA anomaly that we have examined here, suggest that other factors are likely more significant. With this method established, expanding its application to larger, more complete, and more precise datasets becomes manageable. Such efforts have the potential to address fundamental questions about if, how, and when climatic changes impacted past inhabitants by enabling the proposal and evaluation of specific *mechanisms* linking climate and cultural changes.

## Supporting information

S1 Text Cultural history of provence. (DOCX)

S2 Text Data. (DOCX)

S3 Text Methodology. (DOCX)

S4 Text Managing uncertainties. (DOCX)

S5 Text References for SI. (DOCX)

S1 Fig Locations of sites with regard to elevation and slope. (JPG)

S2 Fig Potential pulse productivity of the landscape. (JPG)

S3 Fig Holocene temperature and precipitation in the study area averaged across cultural periods. (JPG)

S1 Table Summary W1 values for landscape and exploited fractions. (DOCX)

S2 Table Comparisons of the W1 means of the exploited fractions for each period to the contemporary landscape (landscape mean of pixelwise means across the period for each pixel), and of each period to every other period. (PDF)

## References

[1] Berger J-F. Les changements climato-environnementaux de l’Holocène ancien et la néolithisation du bassin méditerranéen In: Demoule J-P, Sanchez-Mazas A, Langaney A, editors. La révolution néolithique dans le monde. Paris: CNRS Éditions; 2009 p. 121–144.

[2] Berger J-F, Guilaine J. The 8200 cal BP abrupt environmental change and the Neolithic transition: A Mediterranean perspective. Quaternary International. 2009;200(1–2):31–49.

[3] Carozza L, Berger J-F, Burens A, Marcigny C. Society and environment in Southern France from the 3rd millennium BC to the beginning of the 2nd millennium BC: 2200 BC a tipping point? Tagungen des Landesmuseums für Vorgestchiche Halle. 2015;12:333–62.

[4] Clarke J, Brooks N, Banning EB, Bar-Matthews M, Campbell S, Clare L, et al Climatic changes and social transformations in the Near East and North Africa during the ‘long’ 4th millennium BC: A comparative study of environmental and archaeological evidence. Quaternary Science Reviews. 2016;136:96–121.

[5] Drake BL. The influence of climatic change on the Late Bronze Age Collapse and the Greek Dark Ages. Journal of Archaeological Science. 2012;39(6):1862–1870.

[6] Kaniewski D, Guiot J, Van Campo E. Drought and societal collapse 3200 years ago in the Eastern Mediterranean: a review. Wiley Interdisciplinary Reviews: Climate Change. 2015;6(4):369–382.

[7] Weninger B, Clare L, Rohling E, Bar-Yosef O, Böhner U, Budja M, et al The Impact of Rapid Climate Change on Prehistoric Societies during the Holocene in the Eastern Mediterranean. Documenta Praehistorica. 2009;36:7–59.

[8] Contreras DA. Correlation is Not Enough–Building Better Arguments in the Archaeology of Human-Environment Interactions In: Contreras DA, editor. The Archaeology of Human-Environment Interaction: Strategies for Investigating Anthropogenic Landscapes, Dynamic Environments, and Climate Change in the Human Past. New York: Routledge; 2017 p. 3–22.

[9] Bondeau A, Smith PC, Zaehle S, Schaphoff S, Lucht W, Cramer W, et al Modelling the role of agriculture for the 20th century global terrestrial carbon balance. Global Change Biology. 2007;13(3):679–706.

[10] Contreras DA, Bondeau A, Guiot J, Kirman A, Hiriart E, Bernard L, et al From Paleoclimate Variables to Prehistoric Agriculture: Using a Process-Based Agroecosystem Model to Simulate the Impacts of Holocene Climate Change on Potential Agricultural Productivity in Provence, France Quaternary International 2018;

[11] Contreras DA, Guiot J, Suarez R, Kirman A. Reaching the Human Scale: A Spatial and Temporal Downscaling Approach to the Archaeological Implications of Paleoclimate Data. Journal of Archaeological Science. 2018;93:54–67.

[12] Hodder I, Orton C. Spatial analysis in archaeology. Cambridge: Cambridge University Press; 1976.

[13] Kvamme KL. There and Back Again: Revisiting Archaeological Locational Modeling In: Mehrer M, Wescott KL, editors. GIS and Archaeological Predictive Modeling. Boca Raton: CRC Taylor & Francis; 2006 p. 2–35.

[14] Verhagen P. Case Studies in Archaeological Predictive Modelling. Leiden: Leiden University Press; 2007.

[15] Wheatley D, Gillings M. Spatial Technology and Archaeology: The Archaeological Applications of GIS. London: Taylor & Francis; 2002.

[16] Mehrer M, Wescott K. GIS and Archaeological Predictive Modeling. Boca Raton: CRC Taylor & Francis; 2006.

[17] Wescott KL, Brandon RJ. Practical applications of GIS for archaeologists: A predictive modelling toolkit. London: Taylor & Francis; 2000.

[18] d’Alpoim Guedes JA, Crabtree SA, Bocinsky RK, Kohler TA. Twenty-first century approaches to ancient problems: Climate and society. Proceedings of the National Academy of Sciences. 2016; 10.1073/pnas.1616188113 PMC5187725 27956613

[19] Rohling EJ. Of lakes and fields: A framework for reconciling palaeoclimatic drought inferences with archaeological impacts. Journal of Archaeological Science. 2016;73:17–24.

[20] Blondel J, Aronson J, Bodiou JY, Boeuf G. The Mediterranean region: biological diversity in space and time. 2nd ed Oxford: Oxford University Press; 2010.

[21] Garcia D, Vital J. Dynamiques culturelles de l’âge du Bronze et de l’âge du Fer dans le sud-est de la Gaule. In: Celtes et Gaulois, l’Archéologie face à l`Histoire, 2: la Préhistoire des Celtes. Glux-en-Glenne: Bibracte: Centre archéologique européen; 2006. p. 63–80. (Actes de la table ronde de Bologne-Monterenzio, 28–29 mai 2005).

[22] Vital J. Du Néolithique final au Bronze moyen dans le sud-est de la France: 2200–1450 AV. JC. Cypsela: revista de prehistòria i protohistòria. 2004;15:11–38.

[23] Binder D, Lepère C, Maggi R. Épipaléolithique et Néolithique dans l’arc liguro-provençal: bilan et perspectives de recherche. Bulletin di Musée d’Anthropologie Préhistorique de Monaco, Supplément. 2008;1:49–62.

[24] Binder D. Mesolithic and Neolithic interaction in southern France and northern Italy: new data and current hypotheses In: Price TD, editor. Europe’s first farmers. Cambridge: Cambridge University Press; 2000 p. 117–143.

[25] Guilaine J, Manen C. From Mesolithic to Early Neolithic in the western Mediterranean. Proceedings of the British Academy. 2007;144:21–51.

[26] Buisson-Catil J. Vaucluse préhistorique, Le territoire, les hommes, les cultures et les sites. Le Pontet; 2004.

[27] Ruas M-P, Marinval P. L’alimentation végétale et l’agriculture en France (9000 a.C. - 15e s. p.C.) In: Guilaine J, editor. Pour une archéologie agraire: à la croisée des sciences de l’homme et de la nature. Paris: A. Colin; 1991 p. 409–39.

[28] Gassin B, Ferreira Bicho N, Bouby L, Buxo Capdevila R, Faustino Carvalho A. Variabilité des techniques de récolte et traitement des céréales dans l’occident méditerranéen au Néolithique ancien et moyen: facteurs environnementaux, économiques et sociaux. In: Actes des 7e Rencontres Méridionales de Préhistoire Récentes. 2008 p. 1–23.

[29] Antolín F, Jacomet S, Buxó R. The hard knock life. Archaeobotanical data on farming practices during the Neolithic (5400–2300 cal BC) in the NE of the Iberian Peninsula. Journal of Archaeological Science. 2015;61(C):90–104.

[30] Bogaard A. Neolithic farming in central Europe: an archaeobotanical study of crop husbandry practices. Routledge; 2004.

[31] Bogaard A. The nature of early farming in central and south-east Europe. Documenta Praehistorica. 2004;XXXI:49–58.

[32] Baum T, Nendel C, Jacomet S, Colobran M, Ebersbach R. “Slash and burn” or “weed and manure”? A modelling approach to explore hypotheses of late Neolithic crop cultivation in pre- alpine wetland sites. Vegetation History and Archaeobotany. 2016;1–19.

[33] Jacomet S, Ebersbach R, Akeret O, Antolin F, Baum T, Bogaard A, et al On-site data cast doubts on the hypothesis of shifting cultivation in the late Neolithic (c. 4300–2400 cal. BC): Landscape management as an alternative paradigm. The Holocene. 2016;1–17.

[34] Rösch M, Kleinmann A, Lechterbeck J, Wick L. Botanical off-site and on-site data as indicators of different land use systems: a discussion with examples from Southwest Germany. Vegetation history and archaeobotany. 2014;23(1):121–133.

[35] Magnin F, Martin S. Grandeur et misère de l’analyse malacologique, ou comment discriminer les facteurs climatiques et anthropiques de l’évolution des paysages holocènes. In: Mélanges offerts à Gaetan Congès et Gérard Sauzade. BAP Suppl. 5; 2008 p. 61–73.

[36] Bouby L. L’agriculture dans le bassin du Rhône du Bronze final à l’Antiquité: Agrobiodiversité, économie, cultures. Toulouse: Archives d’écologie préhistorique; 2014.

[37] Marinval P. Des Gaulois aux Gallo-romains, l’agriculture du Midi de la France. Pallas. 2004;64:233–42.

[38] Stika H-P, Heiss AG. Plant cultivation in the Bronze Age In: Harding A, Fokkens H, editors. The Oxford Handbook of the European Bronze Age. Oxford: Oxford University Press; 2013 p. 348–369.

[39] Bouby L. L’économie agricole à l’âge du Bronze en France méridionale. Apports récents de la carpologie. In: Garcia D, editor. L’âge du Bronze en Méditerranée Recherches récentes. 2011. p. 101–14.

[40] Terral J-F. Débuts de la domestication de l’olivier (Olea europaea L.) en Méditerranée nord-occidentale, mise en évidence par l’analyse morphométrique appliquée à du matériel anthracologique. Comptes Rendus de l’Académie des Sciences de Paris. 1997;324(5):417–25.

[41] Garcia D. La Celtique méditerranéenne, Habitats et sociétés en Languedoc et en Provence VIIIe-IIe s. a.C. Arles; 2014.

[42] Garcia D. Dynamiques territoriales en Gaule méridionale durant l’âge du Fer. In: Territoires celtiques Espaces ethniques et territoires des agglomérations protohistoriques d’Europe occidentale. 2002 p. 88–103.

[43] Garcia D, Isoardi D. Variations démographiques et capacités de production des céréales en Celtique méditerranéenne: le rôle de Marseille grecque. In: Grecs et indigènes de la Catalogne à la Mer Noire. 2010 p. 403–24.

[44] Isoardi D. Archéodémographie des sociétés protohistoriques de Sud-Est de la France. Arqueología Espacial. 2010;28:265–84.

[45] Walsh K, Mocci F. Fame and Marginality: The Archaeology of the Montagne Sainte Victoire (Provence, France). American Journal of Archaeology. 2003;107:45–69.

[46] de Beaulieu J-L, Miras Y, Andrieu-Ponel V, Guiter F. Vegetation dynamics in north-western Mediterranean regions: Instability of the Mediterranean bioclimate. Plant Biosystems—An International Journal Dealing with all Aspects of Plant Biology. 2005;139(2):114–26.

[47] Jalut G, Dedoubat JJ, Fontugne M, Otto T. Holocene circum-Mediterranean vegetation changes: Climate forcing and human impact. Quaternary International. 2009;200(1):4–18.

[48] Magny M, Vannière B, Calo C, Millet L, Leroux A, Peyron O, et al Holocene hydrological changes in south-western Mediterranean as recorded by lake-level fluctuations at Lago Preola, a coastal lake in southern Sicily, Italy. Quaternary Science Reviews. 2011;30(19–20):2459–2475.

[49] Vannière B, Power MJ, Roberts N, Tinner W, Carrion J, Magny M, et al Circum-Mediterranean fire activity and climate changes during the mid-Holocene environmental transition (8500–2500 cal. BP). The Holocene. 2011;21(1):53–73.

[50] Berger J-F, Delhon C, Magnin F, Bonté S, Peyric D, Thiébault S, et al A fluvial record of the mid-Holocene rapid climatic changes in the middle Rhone valley (Espeluche-Lalo, France) and of their impact on Late Mesolithic and Early Neolithic societies. Quaternary Science Reviews. 2016;136(C):66–84.

[51] Magny M, Miramont C, Sivan O. Assessment of the impact of climate and anthropogenic factors on Holocene Mediterranean vegetation in Europe on the basis of palaeohydrological records. Palaeogeography, Palaeoclimatology, Palaeoecology. 2002;186:47–59.

[52] Jalut G, Amat AE, Bonnet L, Gauquelin T, Fontugne M. Holocene climatic changes in the Western Mediterranean, from south-east France to south-east Spain. Palaeogeography, Palaeoclimatology, Palaeoecology. 2000;160:255–290.

[53] Frigola J, Moreno A, Cacho I, Canals M, Sierro FJ, Flores JA, et al Holocene climate variability in the western Mediterranean region from a deepwater sediment record. Paleoceanography. 2007;22(2).

[54] Cacho I, Grimalt JO, Canals M, Sbaffi L, Shackleton NJ, Schönfeld J, et al Variability of the western Mediterranean Sea surface temperature during the last 25,000 years and its connection with the Northern Hemisphere climatic changes. Paleoceanography. 2001;16(1):40–52.

[55] Magny M, Combourieu Nebout N, de Beaulieu JL, Bout-Roumazeilles V, Colombaroli D, Desprat S, et al North–south palaeohydrological contrasts in the central Mediterranean during the Holocene: tentative synthe- sis and working hypotheses. Climate of the Past Discussions. 2013;9:1901–67.

[56] Weiss H, Courty M-A, Wetterstrom W, Guichard F, Senior L, Meadow R, et al The Genesis and Collapse of Third Millennium North Mesopotamian Civilization. Science. 1993;261(5124):995–1004. 10.1126/science.261.5124.995 17739617 10.1126/science.261.5124.995

[57] Brooks N. Beyond collapse: climate change and causality during the Middle Holocene Climatic Transition, 6400–5000 years before present. Geografisk Tidsskrift-Danish Journal of Geography. 2012;112(2):93–104.

[58] Magny M, Vannière B, Zanchetta G, Fouache E, Touchais G, Petrika L, et al Possible complexity of the climatic event around 4300–3800 cal. BP in the central and western Mediterranean. The Holocene. 2009;19(6):823–33.

[59] Weinelt M, Schwab C, Kneisel J, Hinz M. Climate and societal change in the western Mediterranean area around 4.2 ka BP In: Meller H, Arz HW, Jung R, Risch R, editors. 2200 BC- A climatic breakdown as a cause for the collapse of the old world? Halle: Landesmuseum für Vorgeschichte; 2015 p. 461–80.

[60] Middleton GD. Nothing Lasts Forever: Environmental Discourses on the Collapse of Past Societies. Journal of Archaeological Research. 2012;20(3):257–307.

[61] Rosen AM. Civilizing climate: social responses to climate change in the ancient Near East. Lanham: Rowman Altamira; 2007.

[62] Drake BL, Blanco-González A, Lillios KT. Regional Demographic Dynamics in the Neolithic Transition in Iberia: Results from Summed Calibrated Date Analysis. Journal of Archaeological Method and Theory. 2017;24(3):796–812.

[63] Bernabeu Aubán J, García Puchol O, Barton M, McClure S, Pardo Gordó S. Radiocarbon dates, climatic events, and social dynamics during the Early Neolithic in Mediterranean Iberia. Quaternary International. 2016;403:201–210.

[64] NASA JPL. NASA Shuttle Radar Topography Mission Global 1 arc second number [Internet]. NASA LP DAAC; 2013 Available from: 10.5067/MEaSUREs/SRTM/SRTMGL1N.003

[65] Guiot J, Kaniewski D. The Mediterranean Basin and Southern Europe in a warmer world: what can we learn from the past? Frontiers in Earth Science. 2015;3(28):1–16.

[66] Currie TE, Bogaard A, Cesaretti R, Edwards N, Francois P, Holden P, et al Agricultural productivity in past societies: Toward an empirically informed model for testing cultural evolutionary hypotheses. Cliodynamics. 2015;6:24–56.

[67] Sitch S, Smith B, Prentice IC, Arneth A, Bondeau A, Cramer W, et al Evaluation of ecosystem dynamics, plant geography and terrestrial carbon cycling in the LPJ dynamic global vegetation model. Global Change Biology. 2003;9(2):161–185.

[68] Fader M, Von Bloh W, Shi S, Bondeau A, Cramer W. Modelling Mediterranean agro-ecosystems by including agricultural trees in the LPJmL model. Geoscientific Model Development. 2015;8(11):3545–3561.

[69] Gorenflo L, Gale N. Population and productivity in the Teotihuacan Valley: changing patterns of spatial association in prehispanic central Mexico. Journal of Anthropological Archaeology. 1986;5(3):199–228.

[70] Seltzer GO, Hastorf C. Climatic Change and Its Effect on Prehispanic Agriculture in the Central Peruvian Andes. Journal of Field Archaeology. 1990;17(4):397–414.

[71] Berger J-F. Hydrological and post-depositional impacts on the distribution of Holocene archaeological sites: The case of the Holocene middle Rhône River basin, France. Geomorphology. 2011;129(3–4):167–182.

[72] Barton CM, Bernabeu J, Aura JE, Garcia O. Land-use dynamics and socioeconomic change: an example from the Polop Alto Valley. American Antiquity. 1999;64(4):609–634.

[73] Bevan A, Crema E, Li X, Palmisano A. Intensities, interactions and uncertainties: some new approaches to archaeological distributions In: Computational approaches to archaeological spaces. New York: Routledge; 2013 p. 27–52.

[74] Thurston TL, Fisher CT, editors. Seeking a Richer Harvest: The Archaeology of Subsistence Intensification, Innovation, and Change. New York: Springer; 2007.

[75] Flohr P, Fleitmann D, Matthews R, Matthews W, Black S. Evidence of resilience to past climate change in Southwest Asia: Early farming communities and the 9.2 and 8.2 ka events. Quaternary Science Reviews. 2016;136(C):23–39.

[76] Halstead P. Two oxen ahead: pre-mechanized farming in the Mediterranean. Malden, MA: Wiley Blackwell; 2014.

[77] Bernard L. ArkeoGIS [Internet]. 2016. Available from: http://arkeogis.org/public

[78] Bernard L, Ertlen D, Schwartz D. ArkeoGIS, Merging Geographical and Archaeological Datas Online In: Giligny F, Djindjian F, Costa L, Moscati P, Robert S, editors. Concepts, methods and tools Proceedings of the 42nd Annual Conference on Computer Applications and Quantitative Methods in Archaeology. Oxford: Archaeopress; 2015 p. 401–6.

[79] Bayliss A. Rolling Out Revolution: Using Radiocarbon Dating in Archaeology. Radiocarbon. 2009;51(1):123–147.

[80] Bayliss A, Bronk Ramsey C, van der Plicht J, Whittle A. Bradshaw and Bayes: Towards a Timetable for the Neolithic. Cambridge Archaeological Journal. 2007;17(Supplement S1):1–28.

[81] Whittle A, Bayliss A. The times of their lives: from chronological precision to kinds of history and change. Cambridge Archaeological Journal. 2007;17(01):21–28.

[82] Barton C, Ullah IT, Heimsath AM. How to Make a Barranco: Modeling Erosion and Land-Use in Mediterranean Landscapes. Land. 2015;4(3):578–606.

[83] Mitasova H, Barton M, Ullah I, Hofierka J, Harmon RS. GIS-Based Soil Erosion Modeling In: Shroder J, Bishop MP, editors. Treatise on Geomorphology, Volume 3 San Diego: Academic Press; 2013 p. 228–258.

[84] Peeters I, Rommens T, Verstraeten G, Govers G, Van Rompaey A, Poesen J, et al Reconstructing ancient topography through erosion modelling. Geomorphology. 2006 8;78(3–4):250–264.

[85] Vermeer JAM, Finke PA, Zwertvaegher A, Gelorini V, Bats M, Antrop M, et al Reconstructing a prehistoric topography using legacy point data in a depositional environment. Earth Surface Processes and Landforms. 2013;39(5):632–645.

[86] Kintigh KW, Altschul JH, Beaudry MC, Drennan RD, Kinzig AP, Kohler TA, et al Grand challenges for archaeology. Proceedings of the National Academy of Sciences. 2014;111(3):879–880. 10.1073/pnas.1324000111 PMC3903258 24449827

[87] Danielisová A, Olševicová K, Cimler R, Machálek T. Understanding the Iron Age Economy: Sustainability of Agricultural Practices under Stable Population Growth In: Wurzer Gabriel, Kowarik Kerstin, Reschreiter Hans, editors. Agent-based Modeling and Simulation in Archaeology. Springer; 2015 p. 183–216.

[88] Kohler TA, Bocinsky RK, Cockburn D, Crabtree SA, Varien MD, Kolm KE, et al Modelling prehispanic Pueblo societies in their ecosystems. Ecological Modelling. 2012;241:30–41.

[89] Kohler TA, Varien MD, editors. Emergence and Collapse of Early Villages: Models of Central Mesa Verde Archaeology. Berkeley, CA: University of California Press; 2012.

[90] Wilkinson TJ, Christiansen JH, Ur JA, Widell M, Altaweel MR. Urbanization within a Dynamic Environment: Modeling Bronze Age Communities in Upper Mesopotamia. American Anthropologist. 2007;109(1):52–68.

[91] R Core Team. R: A Language and Environment for Statistical Computing [Internet]. Vienna, Austria: R Foundation for Statistical Computing; 2016 Available from: https://www.R-project.org/

[92] Hijmans RJ, van Etten J. raster: Geographic data analysis and modeling [Internet]. 2016 Available from: https://CRAN.R-project.org/package=raster

[93] Baddeley A, Rubak E, Turner R. Spatial Point Patterns: Methodology and Applications with R [Internet]. London: Chapman and Hall/CRC Press; 2015 Available from: http://www.crcpress.com/Spatial-Point-Patterns-Methodology-and-Applications-with-R/Baddeley-Rubak-Turner/9781482210200/

[94] Sarkar D. Lattice: Multivariate Data Visualization with R [Internet]. New York: Springer; 2008 Available from: http://lmdvr.r-forge.r-project.org

