# Supplementary material for: Regional paleoclimates and local consequences: Integrating GIS analysis of diachronic settlement patterns and process-based agroecosystem modeling of potential agricultural productivity in Provence (France)
Source: PLoS One. 2018 Dec 12;13(12):e0207622. doi: 10.1371/journal.pone.0207622 (PMC6291104; doi:10.1371/journal.pone.0207622)
Supplement: S1 Text — (DOCX) [file pone.0207622.s001.docx]

**S1. Cultural History of Provence**

The archaeological settlement pattern data are derived from *Patriarche,* the French national archaeological atlas, a continuously updated database that integrates excavation and survey data from diverse sources: <http://www.culturecommunication.gouv.fr/Politiques-ministerielles/Archeologie/Etude-recherche/Carte-archeologique-nationale>.

The *Patriarche* chronology for the mid-late Holocene is inevitably imprecise at regional scales, since any country-wide scheme must suggest that all archaeological periods were synchronous across France; where there is a mismatch in the date ranges assigned to periods we include the *Patriarche* date range in brackets. The resulting imprecision in the settlement data we employ exacerbates the difficulty of matching cultural patterns to environmental ones, but in the absence of independent chronologies anchored in ^14^C or other absolute dating techniques for many sites, represents the best chronological control available.

Within the broad frame of Holocene human occupation of Provence, the transformations of the Neolithic were the consequence of the beginnings of small (25 to 100 m^2^ - 1 or 2 families [1]) sedentary occupations in the lowlands, though such settlements may have been occupied only for relatively short periods (decades to centuries, rather than millennia as in the tell sites of southeastern Europe and Anatolia) [2]. Animal husbandry, based primarily on goats and sheep and involving at least limited transhumance between coastal and higher elevations is evident even early on, appearing by approximately 6500 BP [3], when subsistence was still routinely supplemented by hunting and fishing [4].

The Neolithic Period as a whole was dynamic. For instance, during the Middle Neolithic (MN; 6650 – 5450 BP [6850-6250 BP in *Patriarche*]), agricultural options were expanded by the introduction of hulled barley (*Hordeum vulgare vulgare*) and fava bean (*Vicia faba*) [5]. In the Final Neolithic of Provence (FN; 5450-4050 BP), unprecedented population growth is evidenced by the appearance of new settlements [1,4].

However, this growth was followed by a population decline across the Early Bronze Age (EBA; 4050-3550 BP [4250 – 3450 BP in *Patriarche*]) and Middle Bronze Age (MBA; 3550-3300 BP [3450-3150 in *Patriarche*]). This declining population continued to innovate in subsistence strategies: beginning in the MBA, and particularly in the Late Bronze Age (LBA; 3300-2675 BP [3150-2700 in Patriarche]), hulled barley (*Hordeum vulgare vulgare*) replaced naked barley (*Hordeum vulgare nudum*) [6], while fava and grass pea (*Lathyrus sativus/cicero*) and spelt [*Triticum spelta*]) became more abundant [7], and cultivation of leguminous crops (fava beans [*Vicia faba*], vetch [*Vicia ervilia*], and lentil [*Lens culinaris*]) [6] increased. Growing populations at the end of the Bronze Age were accompanied by some settlement aggregation, supplementing the small settlements similar in scale to Neolithic ones with sites as large as several hundred inhabitants [8], but the majority of settlements were still small. Transhumance between coastal and higher-elevation inland areas, which had likely begun in the Neolithic, increased [9].

The demographic decline at the beginning of the Early Iron Age (EIA; 2675 – 2550 BP) was followed by the founding of the Greek colony of Marseille in 2550 BP. Growth following Marseille’s foundation [10], however, did not preclude a notable demographic decline around the year 2450 BP [11]. During the subsequent two centuries (2550-2350 BP), demographic growth in Provence is evident from both the foundation of new sites and the aggregation of population into larger sites [10]. Demographic growth in EIA Provence, however, was not monotonic: there is a notable demographic decline around the year 2450 BP just before the beginning of the Late Iron Age (LIA; 2400 – 2002 BP), followed around 2200 BP by new population growth marked by sites at high altitudes and commercially-oriented sites along the coast and fluvial axes [11].

During the LIA cultivation of olive increased, while other tree crops (fig (*Ficus carica*) and vine (*Vitis vinifera*)) were introduced [12] and specialized production (e.g., of wine) began. The practice of Mediterranean polycropping also appeared [11], and there is the first clear evidence of fertilization [5], though manuring was likely practiced much earlier [13,14].

Between 2071-2068 BP (121-118 BC), Provence became a Roman province (Transalpine Gaul). Several Celtic towns were abandoned at this time, but others continued to be occupied, and overall population density increased significantly, especially in the lowlands where agricultural exploitation increased.

By the Gallo-Roman Period, small-scale irrigation had probably been practiced for some time, but large-scale irrigation was introduced (cf. [15]), reducing vulnerability to short-term variability in precipitation. These dynamics intensified at the beginning of the second millennium BP (end of the 1st century BC) with the advent of the Roman Empire.
