# Supplementary material for: Regional paleoclimates and local consequences: Integrating GIS analysis of diachronic settlement patterns and process-based agroecosystem modeling of potential agricultural productivity in Provence (France)
Source: PLoS One. 2018 Dec 12;13(12):e0207622. doi: 10.1371/journal.pone.0207622 (PMC6291104; doi:10.1371/journal.pone.0207622)
Supplement: S2 Text — (DOCX) [file pone.0207622.s002.docx]

**S2. Data**

- Guiot and Kaniewski’s [16] dataset is available in the OT-Med data catalog: <http://database.otmed.fr/otmedgeonetwork/srv/api/records/54b9bf34-57ae-45ea-b455-9f90351e538f>. These data are summarized for the study area in Fig 2, and averaged across cultural periods from *Patriarche* in Fig S3.
- The *Patriarche* atlas is detailed online: <http://www.culturecommunication.gouv.fr/Politiques-ministerielles/Archeologie/Etude-recherche/Carte-archeologique-nationale>
- The R code used for downscaling the climate data is available with the publication that describes the methodology in detail [17].
- LPJmL results for W1/W2 and P1/P2 are available in the OT-Med data catalog and in the Dryad Digital Repository (https://datadryad.org):
  - <http://database.otmed.fr/otmedgeonetwork/srv/fre/catalog.search#/metadata/15db8bc2-2b25-4159-94ed-dcf34c103887>
  - [https://doi.org/10.5061/dryad.4rj3ks0](https://doi.org/10.5061/dryad.4rj3ks0)
