# Supplementary material for: Regional paleoclimates and local consequences: Integrating GIS analysis of diachronic settlement patterns and process-based agroecosystem modeling of potential agricultural productivity in Provence (France)
Source: PLoS One. 2018 Dec 12;13(12):e0207622. doi: 10.1371/journal.pone.0207622 (PMC6291104; doi:10.1371/journal.pone.0207622)
Supplement: S3 Text — (DOCX) [file pone.0207622.s003.docx]

**S3. Methodology**

As the data most easily available for locational analysis concern such environmental variables as elevation, slope, aspect, and vegetation cover, these have been most commonly used, engendering criticism (as for use of GIS in archaeology generally) of simplistic environmental determinism. However, in principle social, political, economic, and ideological variables can also be included if they can be expressed spatially, and a growing body of work explores how this may be achieved (e.g., [18–20]).

A key challenge is that of establishing whether site locations are non-random with respect to landscape variables of interest. That is, how probable is it that site locations reflect cognizance of and interest in particular landscape characteristics (rather than selection of areas of the landscape that just happen to have those characteristics, because, for instance, areas with such characteristics are particularly common, or because such characteristics correlate with another variable of interest [cf. [21, 22]])? As Kvamme [23] points out, there are strong analogies with work on species distribution modeling in ecology (cf. [24]), which faces similar analytical and statistical challenges if somewhat distinct explanatory ones.

Debate over the theoretical sufficiency and practical efficacy of locational analysis notwithstanding (cf. [25, 26]), it continues to serve as a valuable tool for assessing the drivers of settlement pattern and the importance of particular spatial variables, whether environmental (e.g., resource distributions) or cultural (e.g. locations of other sites), and the questions that spurred its initial development continue to stimulate researchers (recently, e.g., [21, 27–31]).
