# Supplementary material for: Regional paleoclimates and local consequences: Integrating GIS analysis of diachronic settlement patterns and process-based agroecosystem modeling of potential agricultural productivity in Provence (France)
Source: PLoS One. 2018 Dec 12;13(12):e0207622. doi: 10.1371/journal.pone.0207622 (PMC6291104; doi:10.1371/journal.pone.0207622)
Supplement: S4 Text — (DOCX) [file pone.0207622.s004.docx]

**S4. Managing Uncertainties**

The chronological periods to which the sites in the *Patriarche* database are assigned vary in length from 300 to 1400 years (see Table 2 and Fig 2), and sites often persist through multiple periods (or are reoccupied at intervals spanning multiple periods), meaning that documented sites often apparently lasted for centuries if not millennia. Continuity of occupation in these cases is impossible to assess given the limitations of the data. This persistence may in part reflect slow rhythms of change in settlement patterns, particularly in earlier periods, but indubitably also reflects the imprecision of the chronologies available (this problem has been explored in detail in the archaeological literature (e.g., [32–35]), remaining an issue even where chronological resolution is much finer [cf. [36]]). The likely effect here is to exaggerate settlement density in earlier (longer) periods, as sites whose occupations may not necessarily overlap, though they are broadly contemporary within a given period, are taken as persisting across entire periods. Foundation of new sites and abandonment of existing ones, in addition, can only be dated by *terminus poste quem* and *terminus ante quem*, respectively – for example, a site that is occupied in the EBA but not the MBA may have been abandoned at the transition between the two periods, or at any point during the EBA. The *Patriarche* database itself addresses this problem by flagging some records as of uncertain chronology, and where possible distinguishing between chronological attributions that are broad due to duration of occupation and those that are broad due to dating uncertainty. We have excluded sites whose chronologies are uncertain. However, these measures apply to sites whose occupations span multiple periods and do not address than the problems inherent in lengthy periods.

In order to focus on sites responsive to changing agricultural potential, we filter the site dataset to focus primarily on occupation and agricultural sites, excluding – except where specified for comparative purposes – sites that are exclusively funerary, defensive, ritual, etc. The numbers of samples (Table 1) remain robust for most periods. Where the counts are notably low (for the MBA, and to a lesser degree for the LBA and the EIA), this scarcity itself is telling, but nonetheless makes generalizations about these periods more uncertain (as reflected in the wider confidence intervals for those periods in Figs 3 and 4).

The scale of landscape taphonomy effects has been explored in detail in the nearby Middle Rhone Valley by Berger [37], where detailed surface and subsurface survey and geoarchaeological analysis allowed estimation of ratios of discovered:existing sites that ranged from 1:9 to 1:47, depending on the period. This suggests the possibility of differential preservation of sites from different periods as well as differential preservation of sites in different areas. Uneven survey coverage of the landscape also undoubtedly contributes, and likely can explain, for example, the apparently anomalously sparse settlement of the low hills at the southern edge of the study area.

The MBA decline, recovery in settlement numbers in the LBA, and LIA florescence are evident in both raw and adjusted data, while the EIA slowdown in growth looks, when adjusted for taphonomic effects, more like an actual decline. The effects of the time-averaging of sites across periods should somewhat counterbalance the emphasis that the adjusted data put on the Neolithic Period: as these are the longest intervals, they overemphasize the number of sites, particularly in the Final Neolithic. EBA settlement relative to subsequent periods may also be overemphasized due to long span of the EBA, but those later periods should be broadly comparable to one another.

As noted in Section 5.3, the significance of changing PAgP over any given period would have depended on the dietary prominence of agriculture; declining agricultural yields could have been buffered by other subsistence strategies. Returns from both foraging and pastoralism would also of course be affected by changes in temperature and precipitation, but we have not attempted to model this here. We also have focused (see Section 3) our agricultural modeling on wheat (as a proxy for cereal agriculture more generally) and included peas (as a proxy for pulses more generally) as a comparandum. In principle one could also investigate potential yields for other crops that were locally and regionally important in later periods, e.g., olive and vine (cf. [38] for the inclusion of tree crops in LPJmL under modern conditions).
