# Supplementary material for: Regional paleoclimates and local consequences: Integrating GIS analysis of diachronic settlement patterns and process-based agroecosystem modeling of potential agricultural productivity in Provence (France)
Source: PLoS One. 2018 Dec 12;13(12):e0207622. doi: 10.1371/journal.pone.0207622 (PMC6291104; doi:10.1371/journal.pone.0207622)
Supplement: S5 Text — (DOCX) [file pone.0207622.s005.docx]

**References for SI**

1. Gallay A, Nicod P-Y. Le Néolithique dans les Alpes occidentales. In: Bötsch G, editor. Évolutions biologiques et culturelles en milieu alpin. Actes de l’université d’été 1999. 2000. p. 17–38.

2. Guilaine J, Manen C. From Mesolithic to Early Neolithic in the western Mediterranean. Proceedings of the British Academy. 2007;144:21–51.

3. Walsh K. The archaeology of Mediterranean landscapes: human-environment interaction from the Neolithic to the Roman period. Cambridge University Press; 2014.

4. Buisson-Catil J. Vaucluse préhistorique, Le territoire, les hommes, les cultures et les sites. Le Pontet; 2004.

5. Ruas M-P, Marinval P. L’alimentation végétale et l’agriculture en France (9000 a.C. - 15e s. p.C.). In: Guilaine J, editor. Pour une archéologie agraire : à la croisée des sciences de l’homme et de la nature. Paris: A. Colin; 1991. p. 409–39.

6. Bouby L. L’économie agricole à l’âge du Bronze en France méridionale. Apports récents de la carpologie. In: Garcia D, editor. L’âge du Bronze en Méditerranée Recherches récentes. 2011. p. 101–14.

7. Stika H-P, Heiss AG. Plant cultivation in the Bronze Age. In: Harding A, Fokkens H, editors. The Oxford Handbook of the European Bronze Age. Oxford: Oxford University Press; 2013. p. 348–369.

8. Garcia D, Vital J. Dynamiques culturelles de l’âge du Bronze et de l’âge du Fer dans le sud-est de la Gaule. In: Celtes et Gaulois, l’Archéologie face à l`Histoire, 2: la Préhistoire des Celtes. Glux-en-Glenne: Bibracte: Centre archéologique européen; 2006. p. 63–80. (Actes de la table ronde de Bologne-Monterenzio, 28-29 mai 2005).

9. Garcia D. La Celtique méditerranéenne, Habitats et sociétés en Languedoc et en Provence VIIIe-IIe s. a.C. Arles; 2014.

10. Garcia D. Dynamiques territoriales en Gaule méridionale durant l’âge du Fer. In: Territoires celtiques Espaces ethniques et territoires des agglomérations protohistoriques d’Europe occidentale. 2002. p. 88–103.

11. Garcia D, Isoardi D. Variations démographiques et capacités de production des céréales en Celtique méditerranéenne : le rôle de Marseille grecque. In: Grecs et indigènes de la Catalogne à la Mer Noire. 2010. p. 403–24.

12. Marinval P. Des Gaulois aux Gallo-romains, l’agriculture du Midi de la France. Pallas. 2004;64:233–42.

13. Bogaard A, Fraser R, Heaton THE, Wallace M, Vaiglova P, Charles M, et al. Crop manuring and intensive land management by Europe’s first farmers. In: Proceedings of the National Academy of Sciences. 2013. p. 12589–12594.

14. Poirier N, Nuninger L. Techniques D’amendement Agraire et Témoins Matériels: Pour une Approche Archéologique des Espaces Agraires Anciens. Association d’histoire des sociétés rurales. 2012;38:11–50.

15. Leveau P. Echelles d’anthropisation et archéologie des campagnes de Gaule du Sud à l’époque romaine. Méditerranée. 1998;90(4):17–26.

16. Guiot J, Kaniewski D. The Mediterranean Basin and Southern Europe in a warmer world: what can we learn from the past? Frontiers in Earth Science. 2015;3(28):1–16.

17. Contreras DA, Guiot J, Suarez R, Kirman A. Reaching the Human Scale: A Spatial and Temporal Downscaling Approach to the Archaeological Implications of Paleoclimate Data. Journal of Archaeological Science. 2018;93:54–67.

18. Favory F, Nuninger L, Sanders L. Integration of geographical and spatial archeological concepts for the study of settlement systems. L’Espace géographique. 2012;41:295–309.

19. Howey MCL. Using Multi-criteria Cost Surface Analysis to Explore Past Regional Landscapes: A Case Study of Ritual Activity and Social Interaction in Michigan, AD 1200-1600. Journal of Archaeological Science. 2007;34(11):1830–46.

20. Verhagen P, Nuninger L, Tourneux F-P, Bertoncello F, Jeneson K. Introducing the Human Factor in Predictive Modelling: a Work in Progress. In: Earl G, Sly T, Chrysanthi A, Murrieta-Flores P, Papadopoulos C, Romanowska I, et al., editors. Archaeology in the Digital Era: Papers from the 40th Annual Conference of Computer Applications and Quantitative Methods in Archaeology (CAA), Southampton, 26-29 March 2012. Amsterdam: Amsterdam University Press; 2013. p. 379–88.

21. Jones EE, Ellis P. Multiscalar Settlement Ecology Study of Piedmont Village Tradition Communities, A.D. 1000–1600. Southeastern Archaeology. 2016;35(2):85–114.

22. Kvamme KL. One-sample tests in regional archaeological analysis: new possibilities through computer technology. American Antiquity. 1990;367–381.

23. Kvamme KL. There and Back Again: Revisiting Archaeological Locational Modeling. In: Mehrer M, Wescott KL, editors. GIS and Archaeological Predictive Modeling. Boca Raton: CRC Taylor & Francis; 2006. p. 2–35.

24. Elith J, Leathwick JR. Species distribution models: ecological explanation and prediction across space and time. Annual Review of Ecology, Evolution, and Systematics. 2009;40(1):677–97.

25. Ebert JI. The State of the Art in “Inductive” Predictive Modeling: Seven Big Mistakes (and Lots of Smaller Ones). In: Wescott K, Brandon RJ, editors. Practical applications of GIS for archaeologists: A predictive modelling toolkit. London: Taylor & Francis; 2000. p. 137–43.

26. Verhagen P, Whitley TG. Integrating Archaeological Theory and Predictive Modeling: a Live Report from the Scene. Journal of Archaeological Method and Theory. 2011;19(1):49–100.

27. Bevan A, Crema E, Li X, Palmisano A. Intensities, interactions and uncertainties: some new approaches to archaeological distributions. In: Computational approaches to archaeological spaces. New York: Routledge; 2013. p. 27–52.

28. Kellett LC. Chanka Settlement Ecology: Disentangling Settlement Decision Making during a Time of Risk in the Andean Highlands. In: Kellett LC, Jones EE, editors. Settlement Ecology of the Ancient Americas. London: Routledge; 2017. p. 227–54.

29. Lowerre A. Rural Settlement in England: Analysing Environmental Factors and Regional Variation in Historic Rural Settlement Organisation Using Regression and Clustering Techniques. Portsmouth: English Heritage; 2014.

30. Miller DS, Carmody SB. Colonization After Clovis: Using the Ideal Free Distribution to Interpret the Distribution of Late Pleistocene and Early Holocene Archaeological Sites in the Duck River Valley, Tennessee. Tennessee Archaeology. 2016;8(1–2):78–101.

31. Wachtel I, Zidon R, Garti S, Shelach-Lavi G. Predictive modeling for archaeological site locations: Comparing logistic regression and maximal entropy in north Israel and north-east China. Journal of Archaeological Science. 2018;92:28–36.

32. Bevan A, Conolly J. Multiscalar approaches to settlement pattern analysis. In: Lock G, Monyneaux B, editors. Confronting Scale in Archaeology: Issues of Theory and Practice. New York: Springer; 2006. p. 217–234.

33. Crema ER. Time and probabilistic reasoning in settlement analysis. In: Barceló JA, Bogdanovic I, editors. Mathematics and Archaeology. Boca Raton: CRC Press; 2015. p. 314–334.

34. Dewar RE. Incorporating variation in occupation span into settlement-pattern analysis. American Antiquity. 1991;56(4):604–620.

35. Kintigh KW. Contending with contemporaneity in settlement-pattern studies. American Antiquity. 1994;59(1):143–148.

36. Ortman SG. Uniform Probability Density Analysis and Population History in the Northern Rio Grande. Journal of Archaeological Method and Theory. 2014;23(1):95–126.

37. Berger J-F. Hydrological and post-depositional impacts on the distribution of Holocene archaeological sites: The case of the Holocene middle Rhône River basin, France. Geomorphology. 2011;129(3–4):167–182.

38. Fader M, Von Bloh W, Shi S, Bondeau A, Cramer W. Modelling Mediterranean agro-ecosystems by including agricultural trees in the LPJmL model. Geoscientific Model Development. 2015;8(11):3545–3561.
