# Supplementary figures and images for: Regional paleoclimates and local consequences: Integrating GIS analysis of diachronic settlement patterns and process-based agroecosystem modeling of potential agricultural productivity in Provence (France)
Source: PLoS One. 2018 Dec 12;13(12):e0207622. doi: 10.1371/journal.pone.0207622 (PMC6291104; doi:10.1371/journal.pone.0207622)

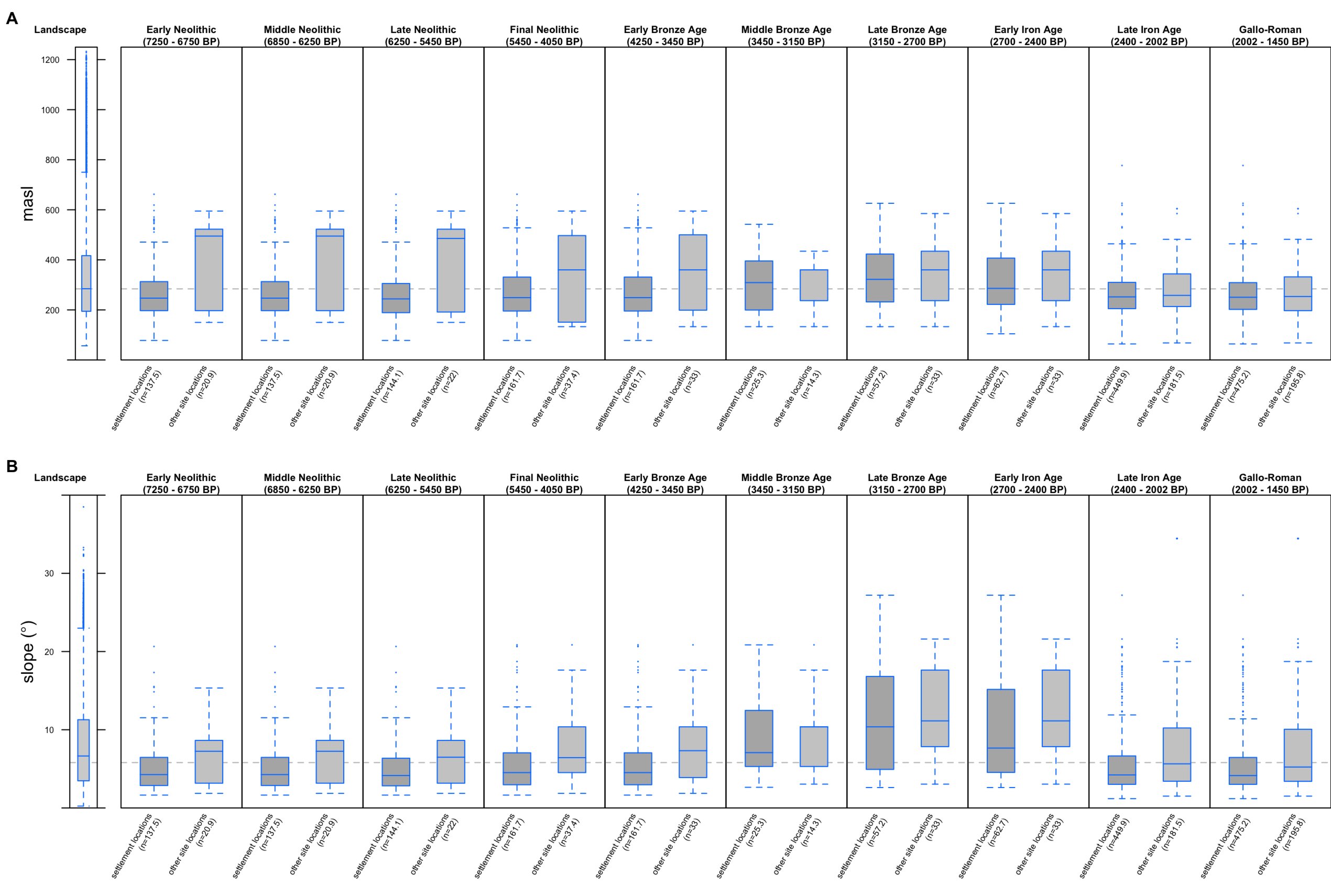

Supplement: S1 Fig — (JPG) [file pone.0207622.s006.jpg]

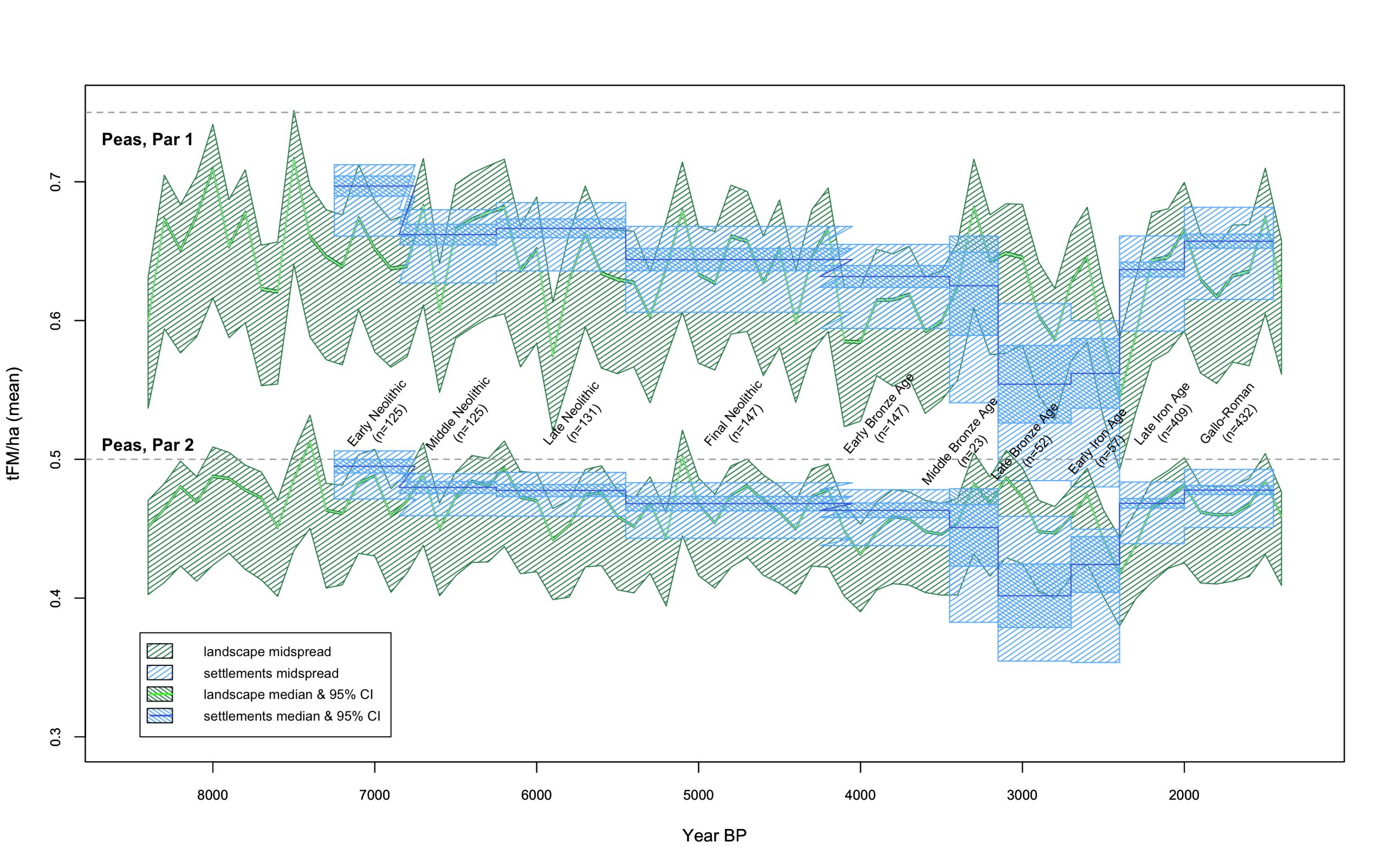

Supplement: S2 Fig — (JPG) [file pone.0207622.s007.jpg]

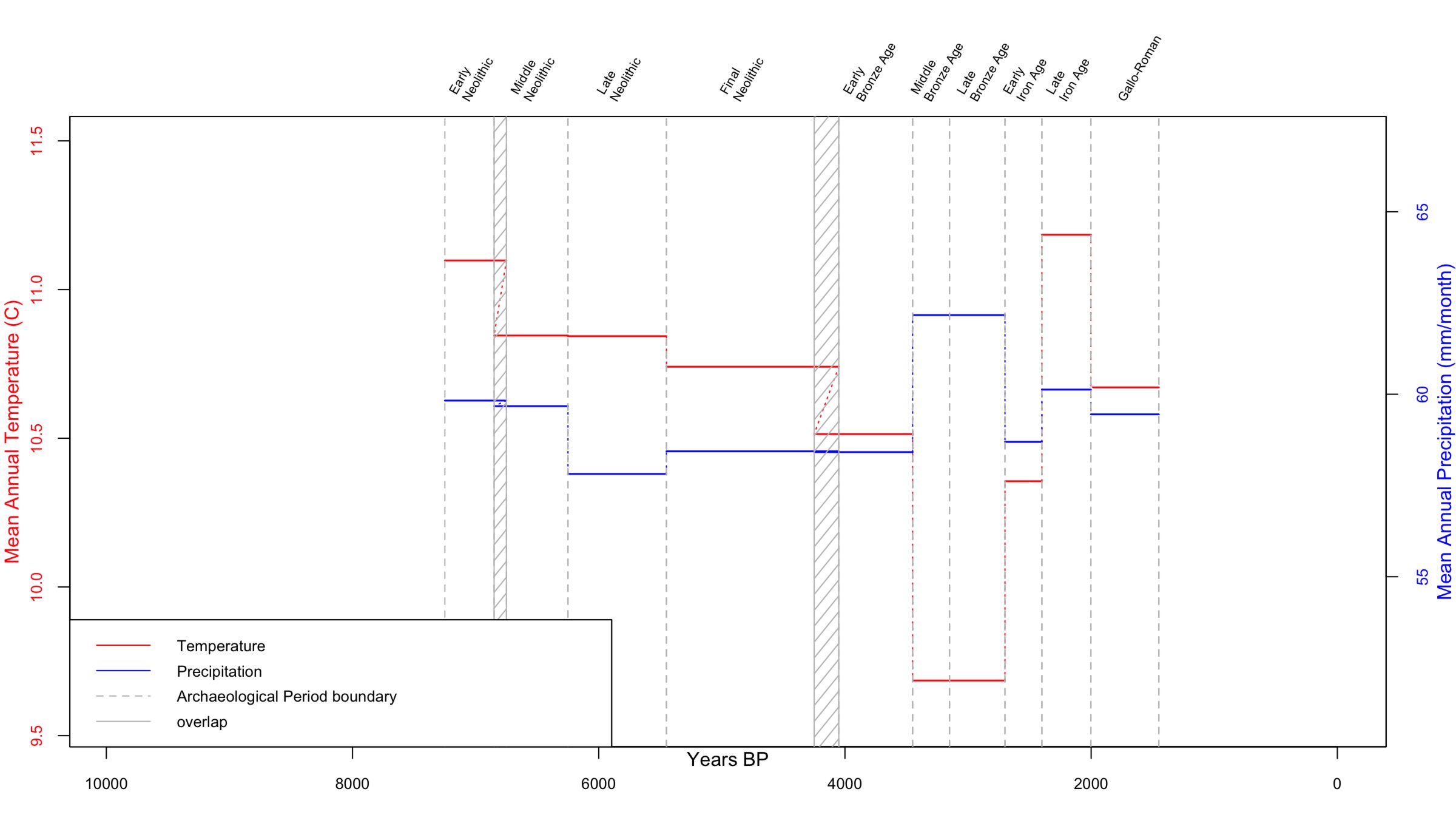

Supplement: S3 Fig — (JPG) [file pone.0207622.s008.jpg]
