# Supplementary material for: Regional paleoclimates and local consequences: Integrating GIS analysis of diachronic settlement patterns and process-based agroecosystem modeling of potential agricultural productivity in Provence (France)
Source: PLoS One. 2018 Dec 12;13(12):e0207622. doi: 10.1371/journal.pone.0207622 (PMC6291104; doi:10.1371/journal.pone.0207622)
Supplement: S1 Table — (DOCX) [file pone.0207622.s009.docx]

Table S1: Summary W1 (high agricultural intensity wheat) values (in tFM/ha) for landscape and exploited fraction

|  | **Landscape** | | |  |  | **Exploited fraction (200m buffer)** | | | **Exploited fraction (500m buffer)** | | | **Exploited fraction (1000m buffer)** | | |
| --- | --- | --- | --- | --- | --- | --- | --- | --- | --- | --- | --- | --- | --- | --- |
| **Year BP** | **median** | **1st quartile** | **3rd quartile** | **Period** | **Number of sites** | **median** | **1st quartile** | **3rd quartile** | **median** | **1st quartile** | **3rd quartile** | **median** | **1st quartile** | **3rd quartile** |
| **8400** | 1.8922 | 1.6977 | 1.9620 |  |  |  |  |  |  |  |  |  |  |  |
| **8300** | 1.9633 | 1.7462 | 2.0286 |  |  |  |  |  |  |  |  |  |  |  |
| **8200** | 1.9798 | 1.7647 | 2.0468 |  |  |  |  |  |  |  |  |  |  |  |
| **8100** | 1.9368 | 1.7441 | 2.0169 |  |  |  |  |  |  |  |  |  |  |  |
| **8000** | 2.0043 | 1.7828 | 2.0679 |  |  |  |  |  |  |  |  |  |  |  |
| **7900** | 1.9325 | 1.7288 | 1.9976 |  |  |  |  |  |  |  |  |  |  |  |
| **7800** | 1.9945 | 1.7785 | 2.0616 |  |  |  |  |  |  |  |  |  |  |  |
| **7700** | 1.9641 | 1.7424 | 2.0283 |  |  |  |  |  |  |  |  |  |  |  |
| **7600** | 1.9422 | 1.7383 | 2.0137 |  |  |  |  |  |  |  |  |  |  |  |
| **7500** | 1.9686 | 1.7791 | 2.0514 |  |  |  |  |  |  |  |  |  |  |  |
| **7400** | 2.1073 | 1.8671 | 2.1744 |  |  |  |  |  |  |  |  |  |  |  |
| **7300** | 1.9909 | 1.7659 | 2.0574 |  |  |  |  |  |  |  |  |  |  |  |
| **7200** | 1.9829 | 1.7709 | 2.0553 | **Early Neolithic**  (7250-6750 BP) |  | 1.3391 | 1.2615 | 1.3732 |  |  |  |  |  |  |
| **7100** | 1.9523 | 1.7620 | 2.0330 |  | 125 |  |  |  | 1.3304 | 1.2694 | 1.3663 | 1.3163 | 1.2590 | 1.3520 |
| **7000** | 1.9966 | 1.7874 | 2.0668 |  |  |  |  |  |  |  |  |  |  |  |
| **6900** | 2.0039 | 1.7872 | 2.0736 |  |  |  |  |  |  |  |  |  |  |  |
| **6800** | 1.9895 | 1.7838 | 2.0636 | **Middle Neolithic**  (6850-6250 BP) |  | 1.3242 | 1.2501 | 1.3550 |  |  |  |  |  |  |
| **6700** | 1.9095 | 1.7270 | 1.9888 |  |  |  |  |  |  |  |  |  |  |  |
| **6600** | 1.9054 | 1.7084 | 1.9760 |  | 125 |  |  |  | 1.3177 | 1.2612 | 1.3510 | 1.3022 | 1.2500 | 1.3323 |
| **6500** | 1.9582 | 1.7413 | 2.0225 |  |  |  |  |  |  |  |  |  |  |  |
| **6400** | 2.0017 | 1.7851 | 2.0674 |  |  |  |  |  |  |  |  |  |  |  |
| **6300** | 2.0202 | 1.8114 | 2.0976 |  |  |  |  |  |  |  |  |  |  |  |
| **6200** | 2.0386 | 1.8125 | 2.1037 | **Late Neolithic**  (6250-5450 BP) |  | 1.4121 | 1.4092 | 1.4151 |  |  |  |  |  |  |
| **6100** | 2.0109 | 1.7863 | 2.0749 |  |  |  |  |  |  |  |  |  |  |  |
| **6000** | 1.9578 | 1.7518 | 2.0276 |  |  |  |  |  | 1.4139 | 1.4138 | 1.4139 | 1.3980 | 1.3972 | 1.3988 |
| **5900** | 1.8753 | 1.6897 | 1.9543 |  | 131 |  |  |  |  |  |  |  |  |  |
| **5800** | 1.9380 | 1.7356 | 2.0129 |  |  |  |  |  |  |  |  |  |  |  |
| **5700** | 1.9637 | 1.7625 | 2.0345 |  |  |  |  |  |  |  |  |  |  |  |
| **5600** | 1.9465 | 1.7513 | 2.0169 |  |  |  |  |  |  |  |  |  |  |  |
| **5500** | 1.9956 | 1.7729 | 2.0618 |  |  |  |  |  |  |  |  |  |  |  |
| **5400** | 1.9464 | 1.7543 | 2.0227 | **Final Neolithic**  (5450-4050 BP) |  | 1.1092 | 0.9940 | 1.2244 |  |  |  |  |  |  |
| **5300** | 1.9216 | 1.7310 | 1.9955 |  |  |  |  |  |  |  |  |  |  |  |
| **5200** | 1.9371 | 1.7373 | 2.0104 |  |  |  |  |  |  |  |  |  |  |  |
| **5100** | 1.9908 | 1.7794 | 2.0577 |  |  |  |  |  |  |  |  |  |  |  |
| **5000** | 1.9667 | 1.7692 | 2.0425 |  |  |  |  |  |  |  |  |  |  |  |
| **4900** | 1.9161 | 1.7288 | 2.0013 |  | 147 |  |  |  | 1.1376 | 1.0389 | 1.2363 | 1.1920 | 1.1030 | 1.2811 |
| **4800** | 2.0392 | 1.8182 | 2.1099 |  |  |  |  |  |  |  |  |  |  |  |
| **4700** | 1.9612 | 1.7675 | 2.0361 |  |  |  |  |  |  |  |  |  |  |  |
| **4600** | 1.9631 | 1.7562 | 2.0326 |  |  |  |  |  |  |  |  |  |  |  |
| **4500** | 1.9833 | 1.7737 | 2.0540 |  |  |  |  |  |  |  |  |  |  |  |
| **4400** | 1.9734 | 1.7696 | 2.0479 |  |  |  |  |  |  |  |  |  |  |  |
| **4300** | 1.9287 | 1.7320 | 1.9993 |  |  |  |  |  |  |  |  |  |  |  |
| **4200** | 2.0262 | 1.7982 | 2.0946 | **Early Bronze Age**  (4250-3450 BP) |  | 1.1119 | 0.9979 | 1.2259 |  |  |  |  |  |  |
| **4100** | 1.8899 | 1.7079 | 1.9711 |  |  |  |  |  |  |  |  |  |  |  |
| **4000** | 1.8225 | 1.6574 | 1.9071 |  |  |  |  |  |  |  |  |  |  |  |
| **3900** | 1.9259 | 1.7331 | 2.0009 |  | 147 |  |  |  | 1.1407 | 1.0432 | 1.2381 | 1.1955 | 1.1078 | 1.2832 |
| **3800** | 1.9586 | 1.7539 | 2.0302 |  |  |  |  |  |  |  |  |  |  |  |
| **3700** | 1.9671 | 1.7585 | 2.0388 |  |  |  |  |  |  |  |  |  |  |  |
| **3600** | 1.9166 | 1.7318 | 1.9989 |  |  |  |  |  |  |  |  |  |  |  |
| **3500** | 1.9121 | 1.7349 | 1.9992 |  |  |  |  |  |  |  |  |  |  |  |
| **3400** | 1.9175 | 1.7260 | 1.9964 | **Middle Bronze Age**  (3450-3150 BP) |  | 1.1115 | 0.9872 | 1.2358 |  |  |  |  |  |  |
| **3300** | 1.9544 | 1.7580 | 2.0285 |  | 23 |  |  |  | 1.1380 | 1.0296 | 1.2463 | 1.1901 | 1.0903 | 1.2899 |
| **3200** | 1.9988 | 1.7826 | 2.0682 |  |  |  |  |  |  |  |  |  |  |  |
| **3100** | 2.0220 | 1.7955 | 2.0873 | **Late Bronze Age**  (3150-2700 BP) |  | 1.1107 | 0.9975 | 1.2239 |  |  |  |  |  |  |
| **3000** | 2.0099 | 1.7953 | 2.0815 |  | 52 |  |  |  | 1.1397 | 1.0433 | 1.2362 | 1.1951 | 1.1089 | 1.2812 |
| **2900** | 1.9396 | 1.7491 | 2.0250 |  |  |  |  |  |  |  |  |  |  |  |
| **2800** | 1.9099 | 1.7168 | 1.9862 |  |  |  |  |  |  |  |  |  |  |  |
| **2700** | 1.9362 | 1.7318 | 2.0019 | **Early Iron Age**  (2700-2400 BP) |  | 1.0889 | 0.9833 | 1.1945 |  |  |  |  |  |  |
| **2600** | 1.9504 | 1.7436 | 2.0147 |  | 57 |  |  |  | 1.1187 | 1.0300 | 1.2074 | 1.1746 | 1.0968 | 1.2524 |
| **2500** | 1.9303 | 1.7194 | 2.0008 |  |  |  |  |  |  |  |  |  |  |  |
| **2400** | 1.8108 | 1.6486 | 1.8973 | **Late Iron Age**  (2400 – 2002 BP) |  | 1.3694 | 1.3359 | 1.3971 |  |  |  |  |  |  |
| **2300** | 1.8779 | 1.7006 | 1.9566 |  | 409 |  |  |  | 1.3589 | 1.3094 | 1.3950 | 1.3510 | 1.2722 | 1.3885 |
| **2200** | 2.0188 | 1.8002 | 2.0860 |  |  |  |  |  |  |  |  |  |  |  |
| **2100** | 1.9935 | 1.7860 | 2.0674 |  |  |  |  |  |  |  |  |  |  |  |
| **2000** | 2.0002 | 1.7956 | 2.0749 | **Gallo-Roman**  (2002 – 1450 BP) |  | 1.3766 | 1.3197 | 1.3900 |  |  |  |  |  |  |
| **1900** | 2.0245 | 1.7987 | 2.0939 |  |  |  |  |  |  |  |  |  |  |  |
| **1800** | 2.0430 | 1.8118 | 2.1128 |  |  |  |  |  | 1.3637 | 1.3066 | 1.3859 | 1.3477 | 1.2659 | 1.3809 |
| **1700** | 1.9535 | 1.7566 | 2.0336 |  | 432 |  |  |  |  |  |  |  |  |  |
| **1600** | 1.9631 | 1.7498 | 2.0318 |  |  |  |  |  |  |  |  |  |  |  |
| **1500** | 2.0389 | 1.8131 | 2.1057 |  |  |  |  |  |  |  |  |  |  |  |
| **1400** | 1.9048 | 1.7167 | 1.9778 |  |  |  |  |  |  |  |  |  |  |  |
