# Supplementary material for: Regional paleoclimates and local consequences: Integrating GIS analysis of diachronic settlement patterns and process-based agroecosystem modeling of potential agricultural productivity in Provence (France)
Source: PLoS One. 2018 Dec 12;13(12):e0207622. doi: 10.1371/journal.pone.0207622 (PMC6291104; doi:10.1371/journal.pone.0207622)
Supplement: S2 Table — (PDF) [file pone.0207622.s010.pdf]

	Early Neolithic			Middle Neolithic			Late Neolithic			Final Neolithic		
	p-value	estimate	95% confidence interva	p-value	estimate	95% confidence interva	p-value	estimate	95% confidence interva	p-value	estimate	95% confidence interva
**Early Neolithic**	NA			0.769	0.004	-0.023 - 0.031	0.9739	0	-0.026 - 0.027	0.1696	0.02	-0.008 - 0.048
**Middle Neolithic**	0.769	-0.004	-0.031 - 0.023	NA			0.7894	-0.004	-0.03 - 0.023	0.2745	0.016	-0.013 - 0.044
**Late Neolithic**	0.9739	0	-0.027 - 0.026	0.7894	0.004	-0.023 - 0.03	NA			0.1713	0.019	-0.008 - 0.047
**Final Neolithic**	0.1696	-0.02	-0.048 - 0.008	0.2745	-0.016	-0.044 - 0.013	0.1713	-0.019	-0.047 - 0.008	NA		
**Early Bronze Age**	0.4567	-0.01	-0.038 - 0.017	0.646	-0.006	-0.034 - 0.021	0.4672	-0.01	-0.037 - 0.017	0.5328	0.009	-0.02 - 0.038
**Middle Bronze Age**	0.0144	-0.116	-0.207 - -0.025	0.0178	-0.112	-0.203 - -0.021	0.0147	-0.116	-0.206 - -0.025	0.0394	-0.096	-0.188 - -0.005
**Late Bronze Age**	0	-0.148	-0.208 - -0.087	0	-0.144	-0.204 - -0.083	0	-0.147	-0.208 - -0.087	1.00E-04	-0.128	-0.189 - -0.067
**Early Iron Age**	0	-0.153	-0.207 - -0.098	0	-0.149	-0.203 - -0.094	0	-0.152	-0.206 - -0.098	0	-0.133	-0.188 - -0.078
**Late Iron Age**	0.1541	0.017	-0.006 - 0.039	0.0784	0.021	-0.002 - 0.043	0.1323	0.017	-0.005 - 0.039	0.0039	0.036	0.012 - 0.061
**Gallo-Roman**	0.4471	0.009	-0.014 - 0.032	0.2712	0.013	-0.01 - 0.036	0.4117	0.009	-0.013 - 0.031	0.0228	0.029	0.004 - 0.053
**landscape**	0	-0.053	-0.072 - -0.034	0	-0.053	-0.072 - -0.034	0	-0.06	-0.078 - -0.041	8.00E-04	-0.037	-0.058 - -0.016

Comparisons of W1 means of the exploited fractions for each period to the contemporary landscape (landscape mean of pixelwise means across the period for each pixel), and of each period to every other period. Each p-value, resulting from a Welch Two Sample t-test, represents the probability that the two sets that intersect in that cell have equivalent means. The estimate (with associated 95% confidence interval) is the difference (in tFM/ha) between the two means that intersect in that cell.

Early Bronze Age			Middle Bronze Age			Late Bronze Age			Early Iron Age			Late Iron Age		
p-value	estimate	95% confidence interval	p-value	estimate	95% confidence interval	p-value	estimate	95% confidence interval	p-value	estimate	95% confidence interval	p-value	estimate	95% confidence interval
0.4567	0.01	-0.017 - 0.038	0.0144	0.116	0.025 - 0.207	0	0.148	0.087 - 0.208	0	0.153	0.098 - 0.207	0.1541	-0.017	-0.039 - 0.006
0.646	0.006	-0.021 - 0.034	0.0178	0.112	0.021 - 0.203	0	0.144	0.083 - 0.204	0	0.149	0.094 - 0.203	0.0784	-0.021	-0.043 - 0.002
0.4672	0.01	-0.017 - 0.037	0.0147	0.116	0.025 - 0.206	0	0.147	0.087 - 0.208	0	0.152	0.098 - 0.206	0.1323	-0.017	-0.039 - 0.005
0.5328	-0.009	-0.038 - 0.02	0.0394	0.096	0.005 - 0.188	1.00E-04	0.128	0.067 - 0.189	0	0.133	0.078 - 0.188	0.0039	-0.036	-0.061 - -0.012
NA			0.0249	0.106	0.014 - 0.197	0	0.137	0.077 - 0.198	0	0.142	0.087 - 0.197	0.0271	-0.027	-0.051 - -0.003
0.0249	-0.106	-0.197 - -0.014	NA			0.5416	0.032	-0.072 - 0.136	0.471	0.036	-0.065 - 0.138	0.0057	-0.133	-0.223 - -0.043
0	-0.137	-0.198 - -0.077	0.5416	-0.032	-0.136 - 0.072	NA			0.9047	0.005	-0.072 - 0.081	0	-0.164	-0.223 - -0.106
0	-0.142	-0.197 - -0.087	0.471	-0.036	-0.138 - 0.065	0.9047	-0.005	-0.081 - 0.072	NA			0	-0.169	-0.222 - -0.116
0.0271	0.027	0.003 - 0.051	0.0057	0.133	0.043 - 0.223	0	0.164	0.106 - 0.223	0	0.169	0.116 - 0.222	NA		
0.1136	0.019	-0.005 - 0.043	0.0086	0.125	0.035 - 0.215	0	0.157	0.098 - 0.216	0	0.161	0.109 - 0.214	0.397	-0.008	-0.026 - 0.01
2.00E-04	-0.04	-0.061 - -0.02	0.1657	0.062	-0.028 - 0.151	0.0016	0.096	0.038 - 0.154	0.002	0.083	0.032 - 0.134	0	-0.05	-0.063 - -0.037

Gallo-Roman			landscape		
p-value	estimate	95% confidence interval	p-value	estimate	95% confidence interval
0.4471	-0.009	-0.032 - 0.014	0	-0.053	-0.072 - -0.034
0.2712	-0.013	-0.036 - 0.01	0	-0.053	-0.072 - -0.034
0.4117	-0.009	-0.031 - 0.013	0	-0.06	-0.078 - -0.041
0.0228	-0.029	-0.053 - -0.004	8.00E-04	-0.037	-0.058 - -0.016
0.1136	-0.019	-0.043 - 0.005	2.00E-04	-0.04	-0.061 - -0.02
0.0086	-0.125	-0.215 - -0.035	0.1657	0.062	-0.028 - 0.151
0	-0.157	-0.216 - -0.098	0.0016	0.096	0.038 - 0.154
0	-0.161	-0.214 - -0.109	0.002	0.083	0.032 - 0.134
0.397	0.008	-0.01 - 0.026	0	-0.05	-0.063 - -0.037
NA			0	-0.057	-0.07 - -0.044
0	-0.057	-0.07 - -0.044	NA		
